# M2 Tumor‐Associated Macrophages‐Derived Exosomal *MALAT1* Promotes Glycolysis and Gastric Cancer Progression

**DOI:** 10.1002/advs.202309298

**Published:** 2024-04-19

**Authors:** Yanzheng Wang, Jiahui Zhang, Hui Shi, Maoye Wang, Dan Yu, Min Fu, Yu Qian, Xiaoxin Zhang, Runbi Ji, Shouyu Wang, Jianmei Gu, Xu Zhang

**Affiliations:** ^1^ Department of Laboratory Medicine School of Medicine Jiangsu University Zhenjiang 212013 China; ^2^ Jiangsu Key Laboratory of Molecular Medicine Medical School of Nanjing University Nanjing 210000 China; ^3^ Department of Clinical Laboratory Medicine Nantong Tumor Hospital/Affiliated Tumor Hospital of Nantong University Nantong 226300 China

**Keywords:** exosomes, gastric cancer, glycolysis, MALAT1, tumor‐associated macrophages

## Abstract

M2‐polarized tumor‐associated macrophages (M2 TAMs) promote cancer progression. Exosomes mediate cellular communication in the tumor microenvironment (TME). However, the roles of exosomes from M2 TAMs in gastric cancer progression are unclear. Herein, it is reported that M2 TAMs‐derived exosomes induced aerobic glycolysis in gastric cancer cells and enhanced their proliferation, metastasis, and chemoresistance in a glycolysis‐dependent manner. It is identified that *MALAT1* (metastasis‐associated lung adenocarcinoma transcript 1) is enriched in M2 TAM exosomes and confirmed that *MALAT1* transfer from M2 TAMs to gastric cancer cells via exosomes mediates this effect. Mechanistically, *MALAT1* interacted with the δ‐catenin protein and suppressed its ubiquitination and degradation by β‐TRCP. In addition, *MALAT1* upregulated HIF‐1α expression by acting as a sponge for miR‐217‐5p. The activation of β‐catenin and HIF‐1α signaling pathways by M2 TAM exosomes collectively led to enhanced aerobic glycolysis in gastric cancer cells. Finally, a dual‐targeted inhibition of *MALAT1* in both gastric cancer cells and macrophages by exosome‐mediated delivery of siRNA remarkably suppressed gastric cancer growth and improved chemosensitivity in mouse tumor models. Taken together, these results suggest that M2 TAMs‐derived exosomes promote gastric cancer progression via *MALAT1*‐mediated regulation of glycolysis. The findings offer a potential target for gastric cancer therapy.

## Graphical abstract

Exosomes are critical regulators of cellular communication in the tumor microenvironment (TME). In this study, Wang et al. reported that M2 tumor‐associated macrophages (M2 TAMs) derived exosomal *MALAT1* (metastasis‐associated lung adenocarcinoma transcript 1) enhanced glycolysis in gastric cancer cells and promoted gastric cancer progression. *MALAT1* exerted this effect via stabilizing δ‐catenin protein to activate the β‐catenin signaling pathway and sponging miR‐217‐5p to upregulate HIF‐1α expression. They further constructed an exosome‐mediated delivery of siRNA system for dual‐targeted inhibition of *MALAT1* in both gastric cancer cells and macrophages, which remarkably suppressed gastric cancer growth and improved chemosensitivity in mouse tumor models.

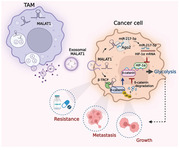

## 1 Introduction

Gastric cancer is one of the leading causes of cancer‐related deaths globally. Despite the recent progress in early diagnosis and treatment, the survival rate of patients with advanced and metastatic disease is still low. The occurrence of metastasis after surgery and therapy resistance remains a major challenge that hinders the improvement of patient survival.^[^ ^1^ ^]^ Therefore, a better understanding of the pathological mechanisms responsible for disease progression in gastric cancer may lead to the design of more effective therapy.

The tumor microenvironment (TME) is a complex ecosystem that consists of distinct cell populations including tumor cells, fibroblasts, and immune cells, among others, and actively participates in tumor development and progression. Tumor‐associated macrophages (TAMs) are a heterogeneous population of myeloid cells present in the TME and are generally divided into M1 and M2 subtypes. Through a re‐education process by tumor, TAMs switch to the pro‐tumor M2 phenotype and collaborate with tumor cells to promote tumor growth and metastasis.^[^ ^2^ ^]^ Previous studies have shown that tumor cells could induce the M2 polarization of TAMs via the secretion of inflammatory cytokines and chemokines, extracellular vesicles, and oncometabolites. In turn, M2 TAMs facilitate tumor growth and metastasis by promoting tumor cell proliferation and EMT (epithelial‐mesenchymal transition), inducing tumor angiogenesis and the formation of a pre‐metastatic niche, and mediating tumor immune suppression.^[^ ^3^ ^]^ Thus, macrophage‐targeting therapeutic strategies have been suggested to complement and synergize with currently available regimens for better cancer therapy.^[^ ^4^ ^]^

Aerobic glycolysis, also known as the Warburg effect, is a hallmark of cancer and plays an important role in tumor progression. The enhanced glycolysis in tumor cells fuels their proliferation, angiogenesis, and metastasis, and also affects therapeutic effect and patient outcomes.^[^ ^5^ ^]^ The glycolytic process of tumor cells is controlled by both intrinsic and external factors and is essential for their malignant behaviors. Recently, there have been studies showing that tumor cells may re‐educate M2 TAMs through lactate, and M2 TAMs promote glycolysis in tumor cells. This process forms a feed‐forward loop to promote malignant tumor progression.^[^ ^6^ ^]^

Exosomes play crucial roles in intercellular communication and are involved in the development and progression of various cancers.^[^ ^7^ ^]^ Exosome‐mediated information exchange orchestrates an important gene expression regulatory network for the dual interplay between tumor cells and non‐tumor cells in the TME, which critically affects tumor growth, metastasis, immunosuppression, and therapy resistance.^[^ ^8^ ^]^ The diverse bioactive cargos in exosomes, including proteins, miRNAs, lncRNAs, and circRNAs, are responsible for their important biological functions. For example, exosomal lncRNAs from different donor cells have been demonstrated to be involved in the pathogenesis, and therapy of many diseases, including cancer.^[^ ^9^ ^]^ Recent studies suggest that exosomes mediate the communications between tumor cells and macrophages.^[^ ^10^ ^]^ However, the detailed mechanism that controls this process has not been fully understood.

In the present study, we demonstrated that M2 TAMs promoted aerobic glycolysis in gastric cancer cells and enhanced their growth, metastasis, and chemoresistance in vitro and in vivo in a glycolysis‐dependent manner. M2 TAMs performed these effects via the release of exosomes that contained a high level of lncRNA *MALAT1*, which stabilized the δ‐catenin protein through lncRNA‐protein interaction and upregulated HIF‐1α via the ceRNA network in gastric cancer cells. Moreover, we developed a therapeutic approach that could potentially achieve dual‐targeted inhibition of *MALAT1* in the TME to improve chemotherapy efficacy.

## 2 Results

### 2.1 M2 TAMs Induce Aerobic Glycolysis in Gastric Cancer Cells and Promote Their Proliferation, Migration, Invasion, and Chemoresistance

We established a classical macrophage polarization induction model by using the THP‐1 cell line (Figure S1A, Supporting Information). FACS analysis confirmed the expression of M2 marker CD206 on M2 TAMs (Figure S1B, Supporting Information). QRT‐PCR results showed that M2 TAMs expressed high levels of arginase1 (Arg1) and immunosuppressive factors TGF‐β, IL‐10, and CCL‐22 compared to M0 macrophages (Figure S1C, Supporting Information).

**Figure 1:**
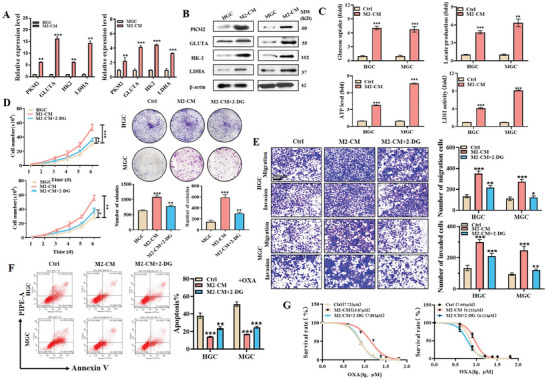
M2‐polarized macrophages induce aerobic glycolysis in gastric cancer cells and promote their proliferation, migration, invasion, and chemoresistance. A,B) QRT‐PCR A) and western blot B) analyses of glycolysis‐related gene expression in gastric cancer cells treated with conditioned medium from M2 macrophages (M2‐CM). C) Glucose uptake, lactate production, ATP level, and LDH activity assays for gastric cancer cells treated with M2‐CM. D,E) Cell counting and colony formation D), transwell migration, and matrigel invasion E) assays for gastric cancer cells treated with M2‐CM in the presence or absence of 2‐DG. Scale bar: 200 µm. F) Flow cytometric analyses on oxaliplatin‐induced apoptosis in gastric cancer cells treated with M2‐CM in the presence or absence of 2‐DG. G) CCK8 assay for IC50 of oxaliplatin in M2‐CM‐treated gastric cancer cells. *p < 0.05, **p < 0.01, ***p < 0.001.

To investigate the effect of M2 TAMs on the glucose metabolism of gastric cancer cells, we collected conditioned medium from M2 TAMs (M2‐CM) and incubated them with gastric cancer cells. QRT‐PCR and western blot results showed that M2‐CM induced the expression of several glycolysis‐related genes including PKM2, GLUTA, HK2, and LDHA in gastric cancer cells (**Figure** **1**A,B). Moreover, M2‐CM treatment enhanced glucose uptake, lactate production, ATP level, and LDH activity in gastric cancer cells (Figure 1C).

Increased aerobic glycolysis promotes cancer growth, metastasis, and therapy resistance. Therefore, we tested the effects of M2‐CM on the malignant progression of gastric cancer. As shown in Figure 1D,E, M2‐CM treatment promoted the proliferation, migration, and invasion abilities of gastric cancer cells; however, pre‐treatment with 2‐DG, a glycolysis inhibitor, significantly reversed this effect. We also observed that M2‐CM treatment decreased the percentage of apoptotic cells induced by oxaliplatin and increased the IC50 (50% inhibitory concentration) of oxaliplatin in gastric cancer cells (Figure 1F,G; Figure S1D, Supporting Information), and again this effect was reversed by co‐incubation with 2‐DG. Taken together, these results indicate that M2 TAMs promote gastric cancer cell proliferation, migration, invasion and chemoresistance through the induction of aerobic glycolysis.

### 2.2 Exosomes from M2 TAMs Facilitate Gastric Cancer Progression by Inducing Aerobic Glycolysis

Exosomes mediate mutual crosstalk between tumor cells and non‐tumor cells and play essential roles in reprogramming the TME to modulate tumor progression.^[^ ^7^ ^]^ We explored whether exosomes from M2 TAMs are responsible for the above‐mentioned effects. GW4869, an exosome inhibitor, was used to suppress exosome production in M2 TAMs and then the CM was collected. As expected, GW4869 treatment inhibited the glycolysis induction and tumor‐promoting effects of M2‐CM on gastric cancer cells (Figure S2, Supporting Information), indicating that exosomes are a key component in M2‐CM that exerted those effects.

**Figure 2:**
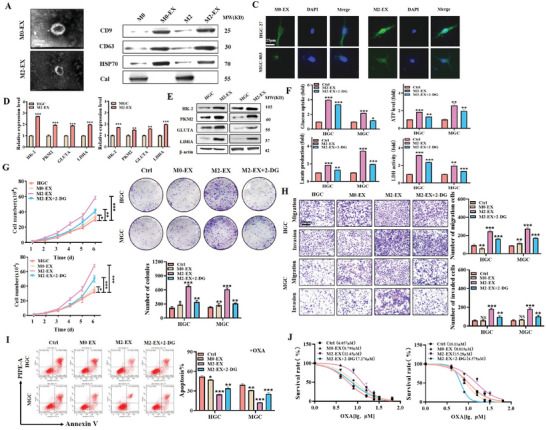
Exosomes from M2‐polarized macrophages induce aerobic glycolysis and promote gastric cancer cell proliferation, migration, and invasion. A) TEM analysis of exosomes from M2 macrophages (M2‐EX). Scale bar: 100 nm. B) Western blot analyses of exosomal proteins in M2‐EX. C) Cellular uptake assay for Dio‐labeled M2‐EX in gastric cancer cells. Scale bar: 25 µm. D,E) QRT‐PCR D) and western blot E) analyses of glycolysis‐related gene expression in gastric cancer cells treated with M2‐EX. F) Glucose uptake, lactate production, ATP level, and LDH activity assays in gastric cancer cells treated with M2‐EX. G,H) Cell counting and colony formation G), transwell migration, and matrigel invasion H) assays for gastric cancer cells treated with M2‐EX in the presence or absence of 2‐DG. Scale bar: 200 µm. I) Flow cytometric analyses of oxaliplatin‐induced apoptosis in gastric cancer cells treated with M2‐EX in the presence or absence of 2‐DG. J) CCK8 assay for IC50 of oxaliplatin in gastric cancer cells treated with M2‐EX. *p < 0.05, **p < 0.01, ***p < 0.001.

We then isolated exosomes from M0 and M2 macrophages and characterized M0‐EX and M2‐EX by TEM, NTA, and western blot (**Figure** **2**A,B; Figure S3A and B, Supporting Information). Cellular uptake assay results demonstrated that M2‐EX could be efficiently internalized by gastric cancer cells (Figure 2C). QRT‐PCR and western blot results showed that, consistent with M2‐CM, M2‐EX treatment also led to the upregulation of glycolysis‐related genes PKM2, GLUTA, HK2, and LDHA in gastric cancer cells (Figure 2D and E). Furthermore, M2‐EX treatment enhanced glucose uptake, lactate production, ATP level, and LDH activity in gastric cancer cells (Figure 2F).

We tested the effects of M2‐EX on gastric cancer cells and found that M2‐EX treatment promoted the proliferation, migration, and invasion abilities of gastric cancer cells. We confirmed that these effects are dependent on the induction of aerobic glycolysis, as pre‐treatment with 2‐DG significantly reduced the roles of M2‐EX (Figure 2G,H). We also observed that M2‐EX treatment reduced oxaliplatin‐induced cell apoptosis and increased the IC50 of oxaliplatin in gastric cancer cells (Figure 2I,G; Figure S3C, Supporting Information), and again this effect was reversed by 2‐DG. Taken together, these results indicate that exosomes from M2 TAMs mimicked their originating cells to promote gastric cancer progression through the induction of aerobic glycolysis.

### 2.3 Exosomal *MALAT1* from M2 TAMs Induces Aerobic Glycolysis and Promotes Gastric Cancer Progression

**Figure 3:**
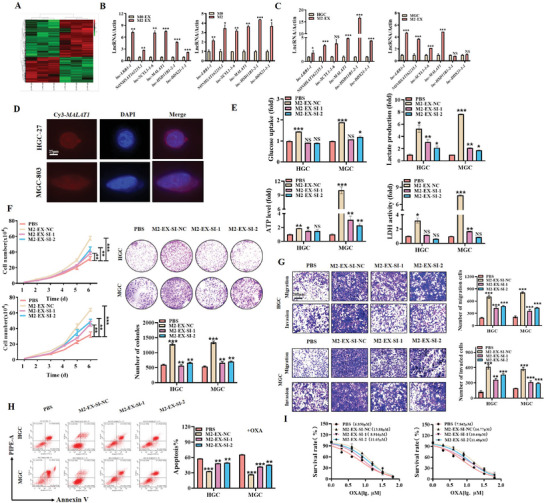
Exosomal *MALAT1* from M2‐polarized macrophages induces aerobic glycolysis and gastric cancer cell proliferation, migration, and invasion. A) Heatmap of RNA‐sequencing analyses of M0‐EX and M2‐EX. B) QRT‐PCR analyses on the top 6 enriched lncRNAs in M2‐polarized macrophages and M2‐EX. C) QRT‐PCR analyses on the top 6 enriched lncRNAs in gastric cancer cells treated with M2‐EX. D) Fluorescent imaging of gastric cancer cells incubated with Cy3‐labeled *MALAT1*‐enriched M2‐EX. Scale bar: 25 µm. E) Glucose uptake, lactate production, ATP level, and LDH activity assays in gastric cancer cells treated with control or *MALAT1*‐depleted M2‐EX. F,G) Cell counting and colony formation F), transwell migration, and matrigel invasion G) assays for gastric cancer cells treated with control or *MALAT1*‐depleted M2‐EX. Scale bar: 200 µm. H) Flow cytometric analyses of oxaliplatin‐induced apoptosis in gastric cancer cells treated with control or *MALAT1*‐depleted M2‐EX. I) CCK8 assay for IC50 of oxaliplatin in control or *MALAT1*‐depleted M2‐EX‐treated gastric cancer cells. *p < 0.05, **p < 0.01, ***p < 0.001.

Exosomes from tumor cells or tumor stromal cells carry bioactive molecules such as lncRNAs to participate in cancer progression. To investigate the specific lncRNAs enriched in M2‐EX, we performed RNA sequencing for M0‐EX and M2‐EX, and compared their lncRNA profiles (**Figure** **3**A). We identified 25 lncRNAs that were enriched in M2‐EX compared to M0‐EX and verified the enrichment of the top 6 lncRNAs in both M2‐polarized macrophages and M2‐EX (Figure 3B). We chose *MALAT1* as the candidate for further study as it has been previously shown to be upregulated in various cancers and associated with cancer progression. We confirmed that M2‐EX treatment led to an upregulation of *MALAT1* in gastric cancer cells (Figure 3C). We further transfected M2‐polarized macrophages with Cy3‐labeled *MALAT1* and collected their derived exosomes to incubate with gastric cancer cells. The intensive presence of Cy3‐labeled *MALAT1* signals in gastric cancer cells indicated that *MALAT1* could be transmitted from M2‐polarized macrophages to gastric cancer cells via exosomes (Figure 3D).

To verify the functional roles of exosomal *MALAT1* from M2‐polarized macrophages, we collected exosomes from M2‐polarized macrophages transfected with si‐Ctrl (SI‐NC) and si‐*MALAT1* (SI‐M1 and SI‐M2), and used them to treat gastric cancer cells. RT‐PCR and western blot results showed that, compared to the SI‐NC group, the upregulation of glycolysis‐related genes PKM2, GLUTA, HK2, and LDHA in gastric cancer cells was reversed in the si‐*MALAT1* groups (Figure S4A and B, Supporting Information). Furthermore, the enhancement of glucose uptake, lactate production, ATP level, and LDH activity in gastric cancer cells by M2‐EX was abolished by *MALAT1* knockdown (Figure 3E). The promotion of gastric cancer cell proliferation, migration, and invasion by M2‐EX was also attenuated by *MALAT1* knockdown (Figure 3F,G). More importantly, we found that the protection against both oxaliplatin‐ and cisplatin‐induced cell apoptosis and the increases in IC50 of gastric cancer cells to oxaliplatin and cisplatin by M2‐EX were reduced by *MALAT1* knockdown (Figure 3H,I; Figures S4C, S5A,B, Supporting Information).

We also validated that *MALAT1* was enriched in exosomes from human peripheral blood monocyte‐derived M2‐polarized macrophages, and aerobic glycolysis was induced to promote gastric cancer cell proliferation, migration, invasion, and chemoresistance (Figures S6 and S7, Supporting Information). We further detected the correlation between *MALAT1* and M2‐polarized macrophages in gastric cancer tissues. The results showed that the upregulated *MALAT1* expression was associated with accumulation of M2‐polarized macrophages in gastric cancer tissues (Figure S8A, and B, Supporting Information). The CM from gastric cancer tissues derived M2 TAMs also enhanced glycolysis in gastric cancer cells but this effect was abolished by *MALAT1* knockdown (Figure S8C–E, Supporting Information). In summary, these results indicate that exosomes from M2‐polarized macrophages induce aerobic glycolysis and promote gastric cancer cell proliferation, migration, and invasion through the transfer of *MALAT1*.

### 2.4 Exosomal *MALAT1* from M2‐Polarized Macrophages Stabilizes δ‐Catenin Protein in Gastric Cancer Cells

**Figure 4:**
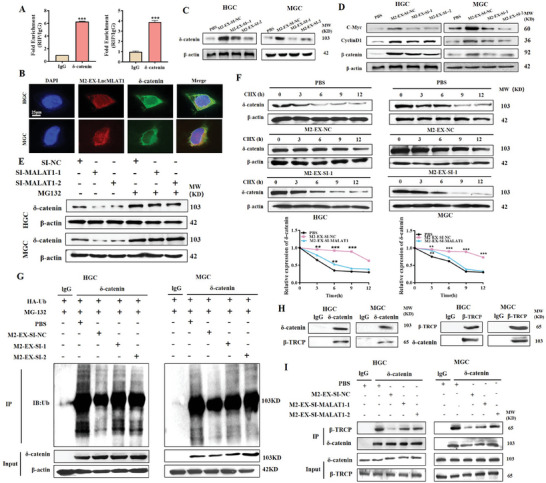
Exosomal *MALAT1* from M2‐polarized macrophages stabilizes δ‐catenin protein. A) RIP assay for the interaction between δ‐catenin protein and *MALAT1*. B) Fluorescent imaging of the co‐localization of Cy3‐labeled *MALAT1* transferred by M2‐EX and δ‐catenin protein in gastric cancer cells. Scale bar: 25 µm. C,D) Western blot assay for δ‐catenin C) and β‐catenin signaling pathway D) proteins in gastric cancer cells treated with control or *MALAT1*‐depleted M2‐EX. E) MG‐132 assay for δ‐catenin protein levels in gastric cancer cells treated with control or *MALAT1*‐depleted M2‐EX. F) CHX assay for the half‐life of δ‐catenin protein in gastric cancer cells treated with control or *MALAT1*‐depleted M2‐EX. G) Western blot assay for the ubiquitination of δ‐catenin protein in gastric cancer cells treated with control or *MALAT1*‐depleted M2‐EX. H) Co‐IP assay for the interaction between δ‐catenin and β‐TRCP in gastric cancer cells. I) Co‐IP assay for the impact of control or *MALAT1*‐depleted M2‐EX on δ‐catenin and β‐TRCP protein interaction. *p < 0.05, **p < 0.01, ***p < 0.001.

To uncover the mechanism for the roles of M2‐EX‐derived *MALAT1* in gastric cancer progression, we predicted the potential interacting proteins of *MALAT1* by using catRAPID software. We found that the δ‐catenin protein is within the candidate list (Figure S9A, Supporting Information) and chose it for further study as we have recently reported that δ‐catenin is critically involved in gastric cancer progression.^[^ ^11^ ^]^ We confirmed the interaction between δ‐catenin and *MALAT1* by RIP assay (**Figure** **4**A). We also found that M2‐EX‐derived *MALAT1*, when internalized by gastric cancer cells, co‐localized with δ‐catenin in the cytosol (Figure 4B). We observed that M2‐EX treatment upregulated δ‐catenin protein expression in gastric cancer cells, while this phenomenon was weakened by *MALAT1* knockdown in M2‐polarized macrophages (Figure 4C). M2‐EX treatment had minimal impact on the mRNA level of δ‐catenin in gastric cancer cells (Figure S9B, Supporting Information). We also confirmed that M2‐EX treatment upregulated β‐catenin and its downstream genes cyclin D1 and c‐Myc (Figure 4D), indicating that M2‐EX activates the β‐catenin pathway via regulation of δ‐catenin.

The results of protein stability assay showed that the low levels of δ‐catenin in gastric cancer cells treated with si‐*MALAT1* M2‐EX could be rescued by MG‐132 treatment (Figure 4E), indicating that *MALAT1* may maintain δ‐catenin protein stability in gastric cancer cells. To confirm this, we performed a cycloheximide (CHX) assay to evaluate the half‐life of δ‐catenin protein in gastric cancer cells treated with si‐NC and si‐*MALAT1* M2‐EX at different times. We found that M2‐EX treatment apparently prolonged the half‐life of δ‐catenin protein, while this effect was not observed after si‐*MALAT1* M2‐EX treatment, indicating that *MALAT1* in M2‐EX is critical for the maintenance of δ‐catenin protein stability (Figure 4F). δ‐catenin protein is regulated at the post‐translation level by the ubiquitin/proteasome system. We thus examined the effect of M2‐EX treatment on the ubiquitination of δ‐catenin protein in gastric cancer cells. IP results showed that, compared to the control group, the levels of ubiquitinated δ‐catenin protein were decreased in the M2‐EX treatment group (Figure 4G); however, the effect of si‐*MALAT1* M2‐EX was compromised, suggesting that *MALAT1* regulates δ‐catenin ubiquitination/degradation. The direct knockdown of *MALAT1* in gastric cancer cells also confirmed that it had no impact on δ‐catenin mRNA level but decreased its protein stability (Figure S9C and D, Supporting Information). Consistent with this, M2‐EX treatment increased the nuclear accumulation of δ‐catenin protein while si‐*MALAT1* M2‐EX had little effect (Figure S9E, Supporting Information).

β‐TRCP (beta‐transducin repeats‐containing proteins) has been identified as a master regulator of δ‐catenin ubiquitination and degradation. We analyzed the potential binding sites for *MALAT1* in δ‐catenin protein and found that there was an overlapping region with the β‐TRCP‐binding site (Figure S9F, Supporting Information). We confirmed the interaction between δ‐catenin and β‐TRCP in gastric cancer cells by co‐IP assay (Figure 4H) and demonstrated that M2‐EX treatment attenuated the interaction between δ‐catenin and β‐TRCP while si‐*MALAT1* M2‐EX did not have this effect (Figure 4I). This finding indicates that *MALAT1* from M2‐EX may interfere with δ‐catenin/β‐TRCP interaction to stabilize δ‐catenin in gastric cancer cells.

### 2.5 Exosomal *MALAT1* from M2‐Polarized Macrophages Upregulates HIF‐1α by Sponging miR‐217‐5p in Gastric Cancer Cells

**Figure 5:**
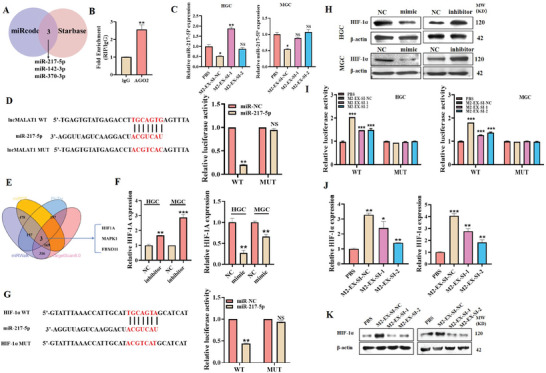
Exosomal *MALAT1* from M2‐polarized macrophages upregulates HIF‐1α by sponging miR‐217‐5p. A) Bioinformatic analyses of potential *MALAT1*‐interacting miRNAs. B) RIP assay for the interaction between AGO2 protein and *MALAT1*. C) QRT‐PCR analyses of miR‐217‐5p expression in gastric cancer cells treated with control or *MALAT1*‐depleted M2‐EX. D) Luciferase assay for the activities of WT and MUT *MALAT1* reporters. E) Bioinformatic analyses of potential target genes of miR‐217‐5p. F) QRT‐PCR analyses of HIF‐1α expression in gastric cancer cells transfected with miR‐217‐5p mimics or inhibitors. G) Luciferase assay for the activities of WT and MUT HIF‐1α reporters. H) Western blot analyses of HIF‐1α protein expression in gastric cancer cells treated with miR‐217‐5p mimics and inhibitors. I) Luciferase assay for the activities of WT and MUT HIF‐1α reporters in gastric cancer cells treated with control or *MALAT1*‐depleted M2‐EX. J,K) QRT‐PCR J) and western blot K) analyses of HIF‐1α protein expression in gastric cancer cells treated with control or *MALAT1*‐depleted M2‐EX. *p < 0.05, **p < 0.01, ***p < 0.001.

Previous studies suggest that exosomal lncRNA may serve as a miRNA sponge to regulate gene expression in the recipient cells. Thus, we searched for the potential miRNAs that might be bound by *MALAT1* by analyzing miRcode and Starbase databases. Three overlapping miRNAs were identified, and we focused on miR‐217‐5p as it has been suggested as a tumor suppressor in various cancers (**Figure** **5**A). We also confirmed the enrichment of *MALAT1* in the AGO2 (argonaute 2) complex by RIP assay (Figure 5A). We observed that M2‐EX treatment decreased miR‐217‐5p expression in gastric cancer cells while *MALAT1*‐depleted M2‐EX had a minimal effect (Figure 5B). According to the potential binding site for miR‐217‐5p in *MALAT1*, we constructed the WT and MUT luciferase reporter vectors for *MALAT1*. The results of luciferase reporter assays showed that miR‐217‐5p overexpression greatly reduced the activity of WT luciferase reporter but did not affect that of the MUT (Figure 5C), indicating that *MALAT1* may serve as a sponge for miR‐217‐5p.

We then predicted the potential target gene that may be regulated by miR‐217‐5p via bioinformatics. The results of Venn plot showed that three overlapping genes were identified, and we chose HIF‐1α for further study as previous studies have shown an essential role of HIF‐1α in cancer progression (Figure 5D). Consistent with that observed in the *MALAT1* luciferase reporter, the results of the luciferase reporter assays showed that miR‐217‐5p overexpression greatly reduced the activity of WT HIF‐1α luciferase reporter but did not affect that of the MUT one (Figure 5E), indicating that HIF‐1α may be a downstream target of miR‐217‐5p. We further confirmed that miR‐217‐5p overexpression increased while miR‐217‐5p inhibition decreased the expression of HIF‐1α in gastric cancer cells (Figure 5D,F). Finally, we tested the effect of M2‐EX on HIF‐1α expression in gastric cancer cells. Luciferase reporter assay results confirmed that M2‐EX treatment increased the activity of WT HIF‐1α luciferase reporter but did not affect that of the MUT one. On the contrary, this regulation was impaired in *MALAT1*‐depleted M2‐EX (Figure 5G). The results of both qRT‐PCR and western blot also showed that M2‐EX treatment upregulated HIF‐1α gene and protein expression while *MALAT1*‐depleted M2‐EX had an attenuated effect (Figure 5H,I). We observed an increase in *MALAT1* expression in gastric cancer tissues compared to normal tissues as well as a moderate positive correlation between CD163 (an indicator of TAM infiltration in tumors) and HIF‐1α gene expression (but not δ‐catenin) in gastric cancer tissues by analyzing TCGA data (Figure S10A–C, Supporting Information). In summary, these data suggest that M2‐EX‐derived *MALAT1* upregulates HIF‐1α by sponging miR‐217‐5p in gastric cancer cells.

### 2.6 Exosomes from M2‐Polarized Macrophages Activate β‐Catenin and HIF‐1α Signaling Pathways to Induce Aerobic Glycolysis and Gastric Cancer Progression

**Figure 6:**
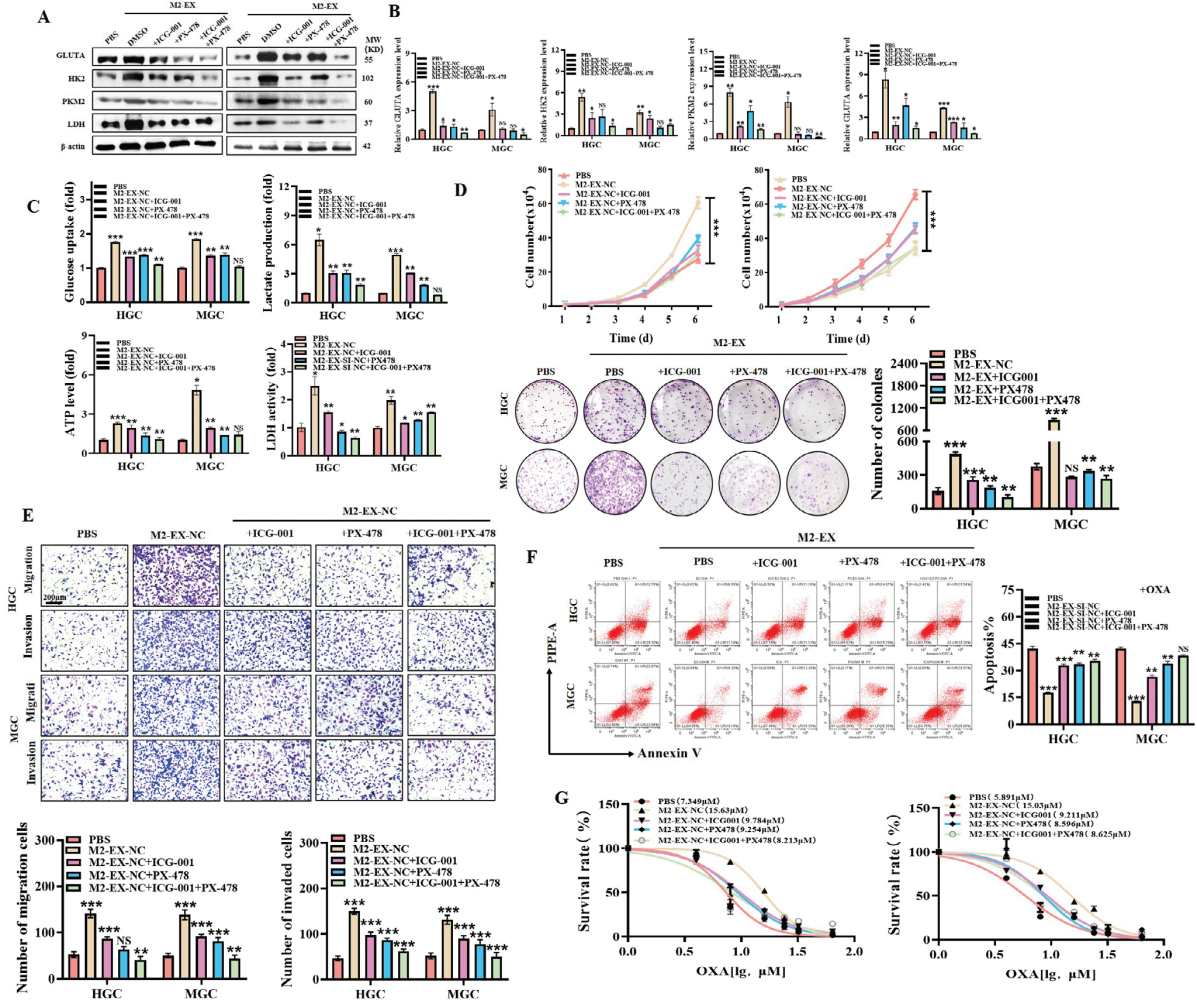
Exosomes from M2‐polarized macrophages activate β‐catenin and HIF‐1α signaling pathways to induce aerobic glycolysis and gastric cancer progression. A,B) Western blot A) and qRT‐PCR B) analyses of glycolysis‐related gene expression in gastric cancer cells treated with M2‐EX in the presence or absence of ICG‐001 and PX‐478. C) Glucose uptake, lactate production, ATP level, and LDH activity assays for gastric cancer cells treated with M2‐EX in the presence or absence of ICG‐001 and PX‐478. D,E) Cell counting and colony formation D), transwell migration, and matrigel invasion E) assays for gastric cancer cells treated with M2‐EX in the presence or absence of ICG‐001 and PX‐478. Scale bar: 200 µm. F) Flow cytometric analyses of oxaliplatin‐induced apoptosis in gastric cancer cells treated with M2‐EX in the presence or absence of ICG001 and PX‐478. G) CCK8 assay for IC50 of oxaliplatin in M2‐EX‐treated gastric cancer cells with the presence or absence of ICG001 and PX‐478. *p < 0.05, **p < 0.01, ***p < 0.001.

We then used the inhibitors for β‐catenin and HIF‐1α signaling pathways to determine the importance of δ‐catenin and HIF‐1α regulation by M2‐EX in promoting glycolysis and gastric cancer progression (Figure S11A and B, Supporting Information). We found that in the presence of β‐catenin inhibitor ICG001 and HIF‐1α inhibitor PX‐478, the upregulation of glycolysis‐related genes PKM2, GLUTA, HK2, and LDHA in gastric cancer cells by M2‐EX was greatly attenuated (**Figure** **6**A,B). In addition, the enhancement of glucose uptake, lactate production, ATP level, and LDH activity in gastric cancer cells by M2‐EX was also impaired by ICG001 and PX‐478 (Figure 6C). Moreover, the promoting effects of M2‐EX on gastric cancer cell proliferation, migration, and invasion were significantly impaired by ICG001 and PX‐478 (Figure 6D,E). More importantly, ICG001 and PX‐478 efficiently inhibited the protective roles of M2‐EX treatment against oxaliplatin‐induced cell apoptosis in gastric cancer cells and decreased their sensitivity to oxaliplatin (Figure 6F,G). Taken together, these results indicate that M2‐EX activates β‐catenin and HIF‐1α signaling pathways to induce aerobic glycolysis and gastric cancer progression.

### 2.7 Targeted Inhibition of *MALAT1* Suppresses the Promotion of Gastric Cancer Progression by M2‐Polarized Macrophages and Improves Chemotherapy Efficacy

**Figure 7:**
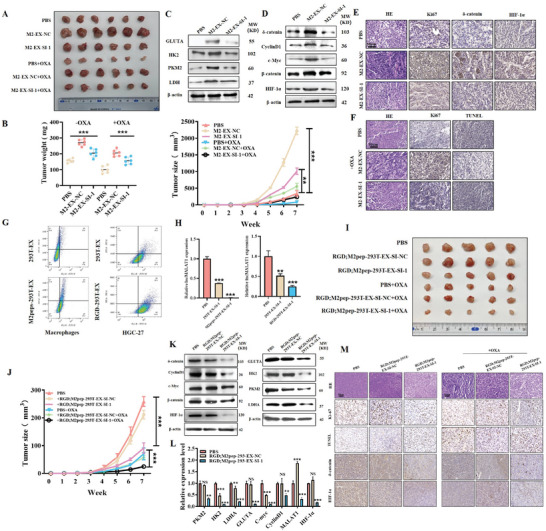
Targeted inhibition of *MALAT1* suppresses the promoting effects of M2‐polarized macrophages on gastric cancer progression and improves chemotherapy efficacy. A) Representative images of tumors in different groups of mouse models as indicated. B) Tumor volumes and growth curves in different groups of mouse models as indicated. C,D) Western blot analyses of the expression of glycolysis‐related proteins C), δ‐catenin, and HIF‐1α D) in different groups of tumors as indicated. E) Immunohistochemical staining of δ‐catenin and HIF‐1α protein expression in different groups of tumors as indicated. Scale bar: 50 µm. F) Ki‐67 staining and TUNEL staining for tumors in different groups of mouse models as indicated. Scale bar: 50 µm. G) Flow cytometric analyses of M2peps‐293T‐EX and RGD‐293T‐EX by macrophages and gastric cancer cells. H) QRT‐PCR analyses of *MALAT1* expression in macrophages and gastric cancer cells treated with *MALAT1* siRNA‐loaded M2peps‐293T‐EX and RGD‐293T‐EX. I,J) Tumor images I), volumes, and growth curves J) in different groups of mouse models as indicated. K) Western blot analyses of δ‐catenin and HIF‐1α protein expression in different groups of tumors as indicated. L) QRT‐PCR analyses of gene expression in different groups of tumors as indicated. M) Immunohistochemical staining of protein expression in different groups of tumors as indicated. Scale bar: 50 µm. *p < 0.05, **p < 0.01, ***p < 0.001.

We performed in vivo animal studies to explore the effect of M2‐EX on gastric cancer progression. Balb/c nude mice were subcutaneously implanted with gastric cancer cells and routinely given intratumoral injections of M2‐EX with or without *MALAT1*. As shown in **Figure** **7**A,B, M2‐EX treatment promoted tumor growth in vivo while *MALAT1*‐depleted M2‐EX had little effect. The results of western blot showed that M2‐EX treatment increased the expression of glycolysis‐related genes (PKM2, GLUTA, HK2, and LDHA) in mouse tumors (Figure 7C). M2‐EX treatment also upregulated δ‐catenin and HIF‐1α expression and activated β‐catenin and HIF‐1α signaling pathways in mouse tumors, whereas these effects were attenuated in mouse tumors treated with *MALAT1*‐depleted M2‐EX (Figure 7D), which was further confirmed by immunohistochemistry results (Figure 7E). In addition, we found that M2‐EX treatment increased the number of metastatic nodules in livers and colon tissues in mice, while *MALAT1* depletion in M2‐EX reversed this effect (Figure S12A and B, Supporting Information). Moreover, we found that the presence of M2‐EX compromised the therapeutic effect of oxaliplatin on mouse tumor models. The depletion of *MALAT1* in M2‐EX reduced its role and improved the therapeutic efficacy of oxaliplatin (Figure 7A,F).

Inspired by the recent development of using exosomes to deliver therapeutic cargos for cancer therapy, we constructed RGD or M2pep‐decorated and si‐*MALAT1*‐encapsulated exosomes (RGD; M2pep‐293T‐EX‐SI‐1) and used the engineered exosomes to target gastric cancer cells and M2 TAMs simultaneously in mouse tumor models. Compared to the unmodified exosomes, RGD; M2pep‐293T‐EX were more efficiently uptaken by gastric cancer cells and M2 TAMs (Figure 7G; Figure S13A and B, Supporting Information) and remarkably led to the inhibition of *MALAT1* expression in both cells (Figure 7H). In vivo animal study results showed that RGD; M2pep‐293T‐EX‐SI‐1 significantly suppressed tumor growth and led to remarkable tumor inhibition when combined with oxaliplatin (Figure 7I,J). Western blot and qRT‐PCR results also showed that RGD; M2pep‐293T‐EX‐SI‐1 treatment reduced δ‐catenin and HIF‐1α expression levels, inactivated β‐catenin pathway, as well as inhibited glycolysis‐related gene expression in mouse tumors (Figure 7K–M). Taken together, these results indicate that RGD; M2pep‐293T‐EX‐SI‐1 efficiently suppresses *MALAT1* expression and suppresses gastric cancer progression.

## 3 Discussion

Exosomes modulate the reciprocal interaction between tumor cells and non‐tumor cells in the TME to promote cancer progression.^[^ ^8^ ^]^ In this study, we reported that M2 TAMs promoted the growth, metastasis, and chemoresistance of gastric cancer in vitro and in vivo via exosomal *MALAT1*, a canonical oncogenic lncRNA. We further illuminated that *MALAT1* exerted these effects through a dual regulatory mechanism. *MALAT1* stabilized δ‐catenin protein and upregulated HIF‐1α gene expression, both of which are master regulators of cancer glycolysis. To the best of our knowledge, this is the first study to show that exosomal *MALAT1* from M2 TAMs could promote cancer progression by inducing glycolysis. Our findings are consistent with two previous studies that explored the function of M2 TAM exosomes in cancer metastasis and chemoresistance, in which *MALAT1* was also identified to be upregulated in M2 TAM exosomes.^[^ ^12^, ^13^ ^]^ Our study further uncovered the biological roles of *MALAT1* upregulation in M2 TAM exosomes and elucidated the underlying mechanisms, adding new information for understanding the interplay between TAMs and tumor cells in the TME and suggesting an important role of exosomal lncRNAs from TME cells in promoting cancer progression.

The versatile roles of M2 TAM exosomes in cancer progression have been widely studied. For instance, exosomes from M2 TAMs are reported to promote tumor growth in lung adenocarcinoma,^[^ ^14^ ^]^ clear‐cell renal cell carcinoma,^[^ ^15^ ^]^ and pancreatic ductal adenocarcinoma.^[^ ^16^ ^]^ In colon cancer, M2 TAM exosomes display high levels of miR‐21‐5p and miR‐155‐5p, promoting cancer cell migration and invasion by decreasing BRG1 expression.^[^ ^17^ ^]^ Exosomes released from M2 TAMs are enriched in miR‐29a‐3p and miR‐21‐5p, which suppresses STAT3 expression in CD4+ T cells and induces Treg/Th17 imbalance, leading to epithelial ovarian cancer progression and metastasis.^[^ ^18^ ^]^ M2 TAM exosomes transmit LINC01232 to enhance the transcription of NBR1 by interacting with E2F2 in glioma cells. The elevated NBR1 binds to and degrades MHC‐I protein on the surface of tumor cells, which in turn leads to tumor cell escape from CD8+ CTL immune attack.^[^ ^19^ ^]^ In ovarian cancer, M2 TAM exosomes promote immune escape via the NEAT/miR‐101‐3p/ZEB1/PD‐L1 axis.^[^ ^20^ ^]^ Moreover, M2 TAM exosomes mediate resistance to radiotherapy, chemotherapy, and immune checkpoint blockade (ICB) therapy in many cancers.^[^ ^21^, ^22^, ^23^, ^24^ ^]^ Previous studies have shown that M2 TAM exosomes promote gastric cancer progression through the transfer of miR‐21, miR‐487a, CRNDE, and apolipoprotein E (ApoE).^[^ ^13^, ^25^, ^26^, ^27^ ^]^ Herein, we reported that exosomes from M2 TAMs transmitted *MALAT1* to gastric cancer cells to promote cancer growth, metastasis, and chemoresistance, while *MALAT1*‐depleted M2 TAM exosomes showed attenuated effects, suggesting that *MALAT1* is one of the key factors in M2 TAM exosomes that promote gastric cancer progression. The importance of *MALAT1* transferred from M2 TAMs to tumor cells in cancer progression deserves further investigation in other cancers.

The regulation of cancer glycolysis by M2 TAM exosomes has been reported. For instance, exosomes from M2 TAMs deliver miR‐222‐3p into laryngeal squamous cell carcinoma (LSCC) cells to suppress PDLIM2 expression, leading to the elevated expression of PFKL and enhanced glycolysis, which accelerates the proliferation of LSCC cells.^[^ ^28^ ^]^ In addition, Xu et al. demonstrated that exosomes from M2 TAMs transmit lncMMPA (M2 macrophage polarization‐associated lncRNA) to hepatocellular carcinoma (HCC) cells and activate glycolysis in HCC cells by regulating the miR‐548s/ALDH1A3 axis and facilitate HCC progression.^[^ ^29^ ^]^ Exosomes may confer chemoresistance through the modulation of cancer glycolysis. Exosomes from M2 TAMs deliver miR‐3679‐5p to lung cancer cells, which downregulates the expression of E3 ligase NEDD4L and stabilizes c‐Myc, leading to elevated glycolysis and chemoresistance.^[^ ^30^ ^]^ Herein, we reported that *MALAT1* was enriched in M2 TAM exosomes and transferred from M2 TAMs to gastric cancer cells to induce glycolysis, showing that M2 TAM exosomes may induce cancer glycolysis through distinct cargos. The biological roles of *MALAT1* in cancer have been well studied. For instance, several studies suggest that the upregulation of *MALAT1* in gastric cancer cells promotes their proliferation, invasion and migration, autophagy, and chemoresistance through a wide spectrum of mechanisms.^[^ ^31^, ^32^, ^33^ ^]^ However, the biological role of *MALAT1* in the TME cells, such as TAMs, is not clear. Recent studies showed that *MALAT1* may have more potent roles in human health and diseases than previously estimated.^[^ ^34^, ^35^, ^36^ ^]^ Thus, our findings establish a previously unknown mechanism for the biological role of *MALAT1* in the TME cells and may also partially explain the dysregulation of *MALAT1* in tumors.

LncRNAs are important regulators of metabolic reprogramming in cancer.^[^ ^37^ ^]^ The regulation of glycolysis by *MALAT1* plays an important role in cancer progression. Pushkar et al. showed that *MALAT1* enhances the translation of the metabolic transcription factor TCF7L2 by upregulating SRSF1 and activating the mTORC1‐4EBP1 axis, contributing to HCC progression.^[^ ^38^ ^]^ In multiple myeloma (MM), *MALAT1* enhances glycolysis in cancer cells via the regulation of miR‐1271‐5p/SOX13 axis.^[^ ^39^ ^]^ In this study, we reported that exosomal *MALAT1* from M2 TAMs stabilized the δ‐catenin protein in gastric cancer cells by suppressing β‐TRCP‐mediated ubiquitination. Previously, Li et al. demonstrated that *MALAT1* stabilizes FOXP3 by binding to its zinc finger and leucine zipper domains, both of which are also interaction domains for E3 ligase STUB1 (STIP1 homology and U‐box containing protein 1), masking the protein‐interacting domains and inhibiting FOXP3 ubiquitination by STUB1.^[^ ^40^ ^]^ Although a similar mechanism may be employed by *MALAT1* to regulate the δ‐catenin/β‐TRCP interaction, the detailed interaction domain was not revealed, which will be determined in future studies. We also found that exosomal *MALAT1* from M2 TAMs upregulated HIF‐1α by sponging miR‐217‐5p. Luo et al. demonstrated that *MALAT1* enhances arsenite‐induced glycolysis in hepatocytes by disassociating the VHL/HIF‐1α interaction and inducing accumulation of HIF‐1α.^[^ ^41^ ^]^ Chen et al. suggested that M2 TAM exosomes transmit HIF‐1α‐stabilizing long noncoding RNA (HISLA) to breast cancer cells to enhance their aerobic glycolysis. Specifically, HISLA inhibits the hydroxylation and degradation of HIF‐1α by blocking the interaction of HIF‐1α with PHD2.^[^ ^12^ ^]^ Collectively, our findings suggest a new mechanism for the regulation of glycolysis by *MALAT1* in cancer progression.

Considering the importance of *MALAT1* in cancer progression, previous studies have developed potential approaches to target *MALAT1* for cancer therapy and verified their therapeutic effects in pre‐clinical studies.^[^ ^42^, ^43^ ^]^ For instance, Wang et al. demonstrated that *MALAT1*/SF2/AR‐v7 axis confers enzalutamide resistance in prostate cancer and the *MALAT1*‐targeting siRNA suppresses enzalutamide‐resistant prostate cancer growth.^[^ ^44^ ^]^ Targeted delivery of *MALAT1* siRNA by nanocomplex also improves the sensitivity of glioblastoma cells to temozolomide.^[^ ^45^ ^]^ Previously, Li et al. constructed an s‐PGEA‐FA/miRNA nanocomplex for targeted delivery of miR‐101 and miR‐217 to silence *MALAT1* in esophageal squamous cell carcinoma (ESCC) cells, achieving effective inhibition of ESCC progression.^[^ ^46^ ^]^ Nicola et al. suggested that targeting *MALAT1* by an LNA‐gapmeR antisense oligonucleotide (ASO) suppresses MM cell proliferation and induces apoptosis in a murine xenograft model of human MM.^[^ ^47^ ^]^ Intriguingly, a recent study demonstrated that *MALAT1* ASO delays primary tumor growth and improves the responses to chemotherapy or ICB therapy in triple‐negative breast cancer (TNBC) mouse models.^[^ ^48^ ^]^ *MALAT1* inhibition decreases the immunosuppressive function of myeloid cells, including TAMs, and increases T‐cell infiltration in the TME. Previously, Chen et al. have developed a mExo‐based delivery system with 7D12 and M2pep modifications to specifically deliver PDL1 siRNAs into M2 TAMs, leading to reprogramming of TAMs and tumor inhibition.^[^ ^49^, ^50^ ^]^ Inspired by the similar strategy, we constructed RGD and M2pep‐engineered exosome to deliver *MALAT1* siRNA and achieved efficient inhibition of *MALAT1* expression in both tumor cells and TAMs, and improved the efficacy of chemotherapy in mouse tumor models, indicating that a rational design of dual‐targeting strategies for both tumor cells and TAMs may contribute to the improved therapeutic efficacy in cancer.^[^ ^51^ ^]^

**Figure 8:**
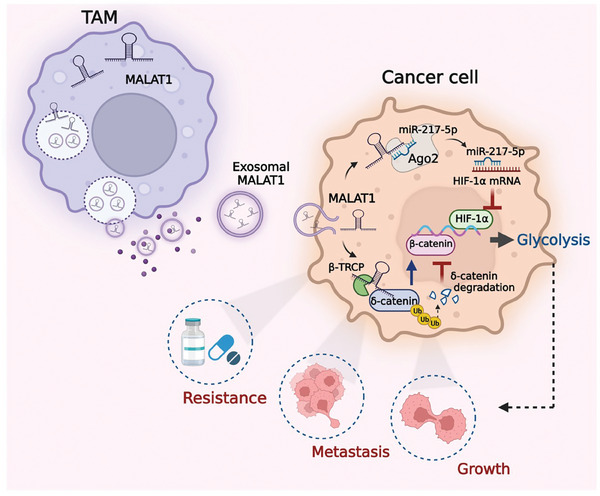
The proposed model for the roles of M2 TAMs in promoting glycolysis and gastric cancer progression.

In conclusion, we identified that M2 TAMs secreted exosomes to transmit *MALAT1* to gastric cancer cells, in which *MALAT1* stabilized the δ‐catenin protein and upregulated HIF‐1α expression, leading to enhanced glycolysis and gastric cancer progression (**Figure** **8**). Our findings unravel a new interplay between M2 TAMs and gastric cancer cells in the TME and suggest a new function of *MALAT1* in gastric cancer progression.

## 4 Experimental Section

### Cell Isolation and Culture

Human gastric cancer cell lines (HGC‐27 and MGC‐803) and human myeloid leukemia mononuclear cells (THP‐1) were purchased from the Cell Bank of the Chinese Academy of Sciences (Shanghai, China). The cells were cultured in RPMI‐1640 medium (Bioind, Israel) supplemented with 10% fetal bovine serum (FBS) at 37 °C in a humid atmosphere (5% CO_2_).

### Human Peripheral Blood Monocyte‐Derived Macrophages

Human peripheral blood samples were collected from healthy volunteers and mononuclear cells (hPBMCs) were isolated and purified by using the Ficoll kit (Serumwerk Bernburg AG, Bernburg, Germany). The cells were then cultured in 6‐well plates and differentiated into macrophages by incubation with recombinant human macrophage colony‐stimulating factor (rhM‐CSF) (100 IU mL^−1^). The medium was changed every 3 days to remove adherent cells, and human monocyte‐derived macrophages could be obtained after 7 days.

### M2 Polarization of Macrophages

THP‐1 cells were treated with 100 ng mL^−1^ of phorbol‐12‐myristate‐13‐acetate (PMA) (Millipore, Shanghai, China) for 24 h to differentiate into macrophage‐like cells. Then, the differentiated macrophages were incubated with IL‐4 (20 ng mL^−1^; Peprotech, Suzhou, China) and IL‐13 (20 ng mL^−1^; Peprotech) for 48 h to induce M2 polarization.

### Cell Transfection

The designed shRNAs, miRNA mimics, and inhibitors were synthesized by GenePharma (Suzhou, China) and transfected into the cells for 48 h with Lipofectamine 2000 (Invitrogen, Shanghai, China) according to the manufacturer's instructions.

### Exosome Extraction and Identification

The conditioned medium was centrifuged at 300 × g for 15 min, 2000 × g for 15 min, and 10 000 × g for 30 min and then filtered through a 100 KD ultrafiltration centrifugal tub, followed by ultrafiltration at 100 000 × g for 70 min (Beckman Coulter, Shanghai, China) twice to collect exosomes. The properties of exosomes were characterized by using transmission electron microscopy (TEM), nanoparticle‐tracking analysis (NTA), and Western blot.

### RNA Sequencing

Total RNA in exosomes were extracted by using miRNeasy Serum/Plasma Kit (Qiagen, Shanghai, China). RNA library construction and sequencing were performed by RiboBio (Guangzhou, China). The cDNA library was sequenced on Illumina Hiseq 2500 (Illumina, Shanghai, China). Illumina analysis software was used to collect the raw reads from sequencing.

### Glucose Consumption and Lactate Production Assays

Cell culture medium was collected 24 h after treatment for the measurement of lactate concentration and glucose consumption. Glucose uptake and lactate production in the medium were detected by glucose uptake assay kit and lactate assay kit (Applygen, Shanghai, China) according to the manufacturer's instructions. The results were normalized to the total cell number of each sample.

### Measurement of Lactate Dehydrogenase Activity and Adenosine Triphosphate Level

The ATP level and cellular LDH activity of transfected cells were assessed by ATP assay kit (Beyotime, Shanghai, China) and Lactate dehydrogenase assay kit (Jiancheng Bio, Nanjing, China), respectively.

### Luciferase Reporter Assay

Gastric cancer cells were seeded at 1 × 10^4^ cells well^−1^ in 24‐well plates and cultured overnight. Wild‐type (WT) and mutant (MUT) luciferase reporter vectors for *MALAT1* and HIF1α were constructed and then co‐transfected with miR‐217‐5p mimics or negative control (NC) into the cells. After transfection for 48 h, the luciferase activity was determined by using a Dual‐Luciferase Reporter Assay System (Promega, Beijing, China) according to the supplier's protocol. All experiments were performed in triplicate.

### Flow Cytometric Analysis

Annexin V‐FITC Apoptosis Detection kit (Vazyme, Nanjing, China) was used to detect cell apoptosis. The treated cells were collected and re‐suspended in 100 µL 1 × binding buffer and incubated with 5 µL Annexin V‐fluorescein isothiocyanate (FITC) at room temperature for 10 min in dark conditions, followed by further incubation with 5 µL propidium iodide (PI) for another 10 min. The percentage of apoptotic cells was measured in a BD CytoFLEX flow cytometer.

### Cell Counting Kit‐8 (CCK‐8) and Colony Formation Assays

For the CCK‐8 assay, the cells were seeded in a 96‐well plate at 2 × 10^3^ cells well^−1^. After 24 h, 10 µL of CCK‐8 reagent (Vazyme) was added to the cells and incubated for 2 h. The OD value of cells in each well at 450 nm was measured at different time points. Cells were plated at a density of 1×10^3^ cells well^−1^ in a 6‐well plate for the colony formation assay with medium change at 3‐day intervals for 9 days. The colonies were fixed in 4% paraformaldehyde for 20 min and then stained with crystal violet for another 20 min at room temperature. The experiment was performed in triplicates.

### Transwell Migration and Matrigel Invasion Assays

Cells suspended in serum‐free medium were seeded in the upper chamber (Corning Life Science, Suzhou, China) with or without Matrigel (BD Biosciences) and the lower chamber was filled with a complete medium. The 6‐well plate was placed at 37 °C in a 5% CO_2_ incubator for 48 h. After that, the chambers were fixed with 4% paraformaldehyde and stained via crystal violet for microscopic observation.

### RNA Fluorescence In Situ Hybridization

Fluorescence in situ hybridization (FISH) kit and *MALAT1* probes were provided by GenePharma. Cells were seeded in a 12‐well plate with slides for 24 h. Then, cells were fixed in 4% paraformaldehyde for 15 min at room temperature and incubated with a FISH probe in a hybridization buffer at 73 °C for 5 min. The hybridization was incubated at 37 °C overnight. Finally, the slides were washed, dehydrated, and stained with DAPI. The images were acquired by using a fluorescent microscope.

### Co‐Immunoprecipitation

Protein A/G agarose conjugated with normal IgG or anti‐δ‐catenin antibody was mixed with pre‐cleaned cell lysates at 4 °C overnight. After washing with the lysis buffer, the proteins were eluted for Western blot analysis.

### Immunofluorescence and Immunohistochemistry

For immunofluorescence analysis, cells were fixed with 4% paraformaldehyde for 15 min and permeabilized with 0.1% Triton‐X‐100 for 20 min. Cells were blocked with 5% bovine serum albumin (BSA) for 1 h at room temperature and then incubated with primary antibody overnight at 4 °C. Subsequently, the cells were washed and stained with Hoechst 33 342 (Sigma, Shanghai, China). For immunohistochemical analysis, tumor tissues were embedded in paraffin and then cut into 5 µm‐thick slices. After dewaxing and rehydrating, endogenous peroxidase was inactivated by using 3% H_2_O_2_ diluted in methanol for 30 min. The slices were blocked with 5% BSA for 1 h at room temperature and then incubated with primary antibodies against δ‐catenin and HIF‐1α at 4 °C overnight. After incubation with the secondary antibody, the slices were stained with 3,3‐diaminobenzidine substrate (DAB) and counterstained with hematoxylin.

### Quantitative Real‐Time PCR and Western Blot

TRIzol reagent (Invitrogen) was used to extract total RNA. Reverse transcription was carried out by using HiScript III 1st Strand cDNA Synthesis Kit (Vazyme). The mRNA levels were determined by qRT‐PCR with the SYBR green system. For the detection of miRNAs, miRNA‐specific primers were used to obtain cDNA by using the miRNA 1st Strand cDNA Synthesis Kit (Vazyme). β‐actin was used as the internal control. The primer sequences were listed in the Table S1 (Supporting Information). Total protein was extracted from the cells using RIPA buffer and determined using the BCA Protein Assay Kit (Vazyme). Total proteins were separated by SDS‐PAGE gels and then transferred to PVDF membranes (Millipore). The membranes were blocked and incubated with specific primary antibodies at 4 °C overnight, followed by incubation with secondary antibodies at room temperature. β‐actin was used as the internal control. The protein bands were visualized by the ECL detection system.

### Preparation of RGD;M2pep‐Engineered Exosomes

293 T cells were transfected with si‐NC and si‐*MALAT1* for 24 h and then cultured in DMEM medium containing 10% exosome‐free FBS. After 48 h, cell culture supernatant was collected, and exosomes were purified as previously described. Exosomes from the 293T‐SI‐NC (293T‐EX‐SI‐NC) and 293T‐SI‐*MALAT1* (293T‐EX‐SI‐*MALAT1*) groups were incubated with 50 µg of DSPE‐PEG‐RGD and 50 µg of DSPE‐PEG‐M2pep (Ruixi Biotechnology, China) at 37 °C for 30 min to form RGD;M2pep‐293T‐EX‐SI‐NC and RGD;M2pep‐293T‐EX‐SI‐*MALAT1*, respectively.

### In Vivo Mouse Xenograft Tumor Model

The animal experiments were approved by the Animal Use and Care Committee of Jiangsu University. 4–6 weeks old male BALB/c nude mice were randomly divided into six groups (n = 5 per group) and maintained in a specific pathogen‐free environment. HGC‐27 cells (3×10^6^ cells) suspended in 100 µL PBS were injected subcutaneously into the mice and tumor growth was monitored every 3 days. Tumor volume was calculated by using the formula: Volume = (width^2^× length)/2). When tumor size reached 100 mm^3^, M2 TAMs derived exosomes (M2‐EX) (control or *MALAT1*‐depleted) was intratumorally injected into the tumor. For chemotherapy, the mice were given a peritoneal injection of oxaliplatin (20 mg k^−1^g body weight). After 6 weeks, the subcutaneous tumors were removed and weighed. For tumor metastasis, HGC‐27 cells (5×10^6^ cells) and M2‐EX (control or *MALAT1*‐depleted) were peritoneally injected into the mice. After 8 weeks, the mice were killed, and the livers and colon tissues were removed for histologic examination. For in vivo anti‐tumor effects of engineered exosomes, the engineered exosomes (100 µg in 100 µL PBS) were injected into the mice through the tail vein every 3 days for a total of 5 injections. For combined therapy, the mice were given a peritoneal injection of oxaliplatin (20 mg k^−1^g body weight) every 7 days for a total of 3 injections. A week after the final injection of engineered exosomes, the mice were sacrificed, and the tissues were collected for further analyses.

### Statistical Analysis

Data are expressed as the mean ± SD and statistical analysis was carried out by using Prism 7.0 (GraphPad, USA). Student's two‐tailed *t*‐test or one‐way ANOVA were used for comparisons and *P*<0.05 was considered as statistically significant.

## Conflict of Interest

The authors declare no conflict of interest.

## Author Contributions

Y.W., J.Z., and H.S. contributed equally to this work. Y.W., J.G., and X.Z. performed conception and design. Y.W., M.W., J.Z., H.S., J.Z., X.D., and D.Y. performed development of methodology. M.F., Y.Q., X.Z., and R.J. performed acquisition of data. Y.W., M.W., J.G., and X.Z. performed analysis and interpretation of data. Y.W., S.W., J.G., and X.Z. wrote, reviewed, and revision of the manuscript. J.G. and X.Z. performed administrative, technical, or material support. X.Z. performed study supervision.

## Supporting information

Supporting Information

## Data Availability

The data that support the findings of this study are available on request from the corresponding author. The data are not publicly available due to privacy or ethical restrictions.
