# Supplementary material for: M2 Tumor‐Associated Macrophages‐Derived Exosomal MALAT1 Promotes Glycolysis and Gastric Cancer Progression
Source: Adv Sci (Weinh). 2024 Apr 19;11(24):2309298. doi: 10.1002/advs.202309298 (PMC11199979; doi:10.1002/advs.202309298)
Supplement: Supplementary file 1 — Supporting Information [file ADVS-11-2309298-s001.pdf]

ADVANCED SCIENCE
Open Access

Supporting Information

for *Adv. Sci.*, DOI 10.1002/advs.202309298

# M2 Tumor-Associated Macrophages-Derived Exosomal *MALAT1* Promotes Glycolysis and Gastric Cancer Progression

*Yanzheng Wang, Jiahui Zhang, Hui Shi, Maoye Wang, Dan Yu, Min Fu, Yu Qian, Xiaoxin Zhang, Runbi Ji, Shouyu Wang, Jianmei Gu* and Xu Zhang**

## Supplementary Materials

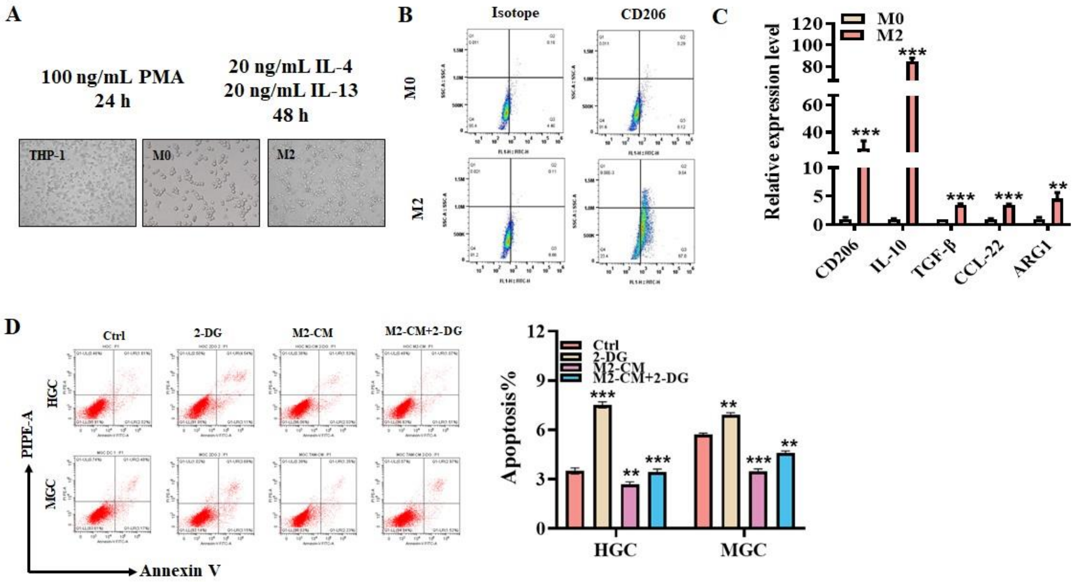


**Supplementary Figure 1. Induction and characterization of M2-polarized macrophages**

**A.** The procedure of inducing macrophage-like cells and M2-polarized macrophages. **B.** Flow cytometric analyses of CD206 expression in M2-polarized macrophages. **C.** QRT-PCR analyses of arginase1, TGF-β, IL-10, and CCL-22 expression in M2-polarized macrophages. **D.** Flow cytometric analyses of apoptosis in gastric cancer cells treated with M2-CM in the presence or absence of 2-DG. $**P< 0.01$, $***P<0.001$.

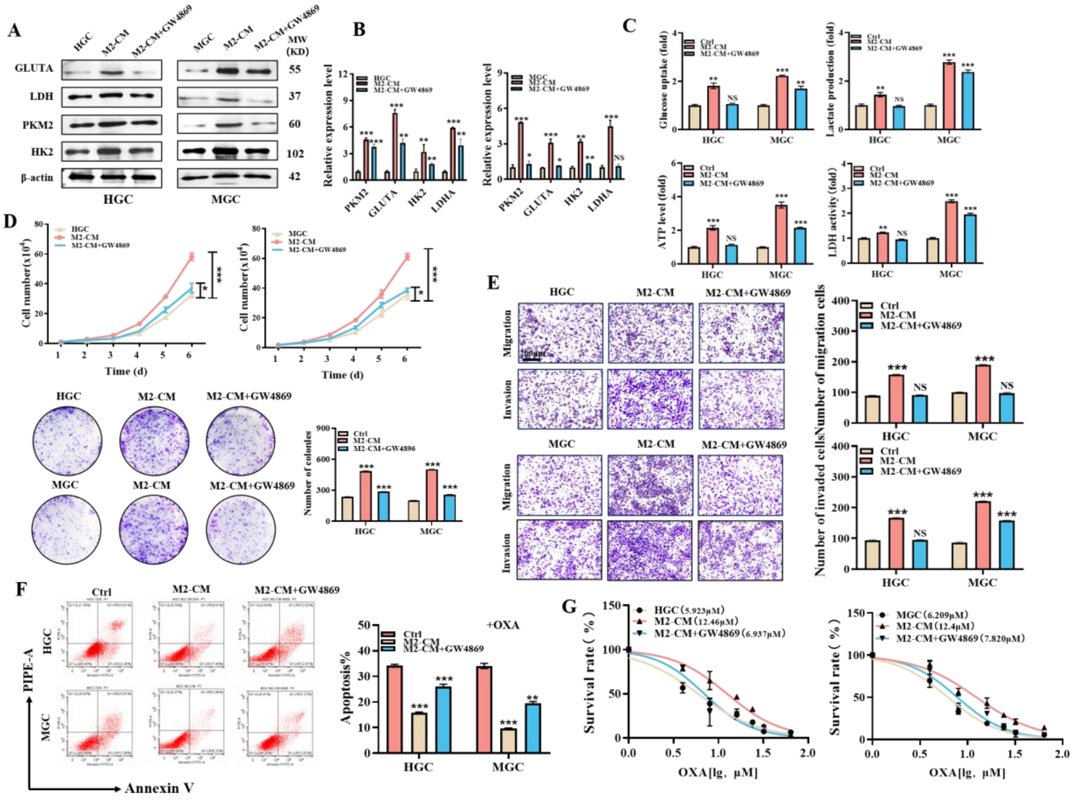


**Supplementary Figure 2. GW4869 inhibits the effects of M2-CM on gastric cancer cell glycolysis**

**A-B.** QRT-PCR (A) and Western blot (B) analyses of glycolysis-related gene expression in gastric cancer cells treated with conditioned medium from M2-CM in the presence or absence of GW4869. **C.** Glucose uptake, lactate production, ATP level, and LDH activity assays for gastric cancer cells treated with M2-CM in the presence or absence of GW4869. **D-E.** Cell counting and colony formation (D), transwell migration, and Matrigel invasion (E) assays for gastric cancer cells treated with M2-CM in the presence or absence of GW4869. Scale bar: 200μm. **F.** Flow cytometric analyses of oxaliplatin-induced apoptosis in gastric cancer cells treated with M2-CM in the presence or absence of GW4869. **G.** CCK8 assay for IC50 of oxaliplatin in M2-CM-treated gastric cancer cells with the presence or absence of GW4869. **P*<0.05, ***P*< 0.01, ****P*<0.001.

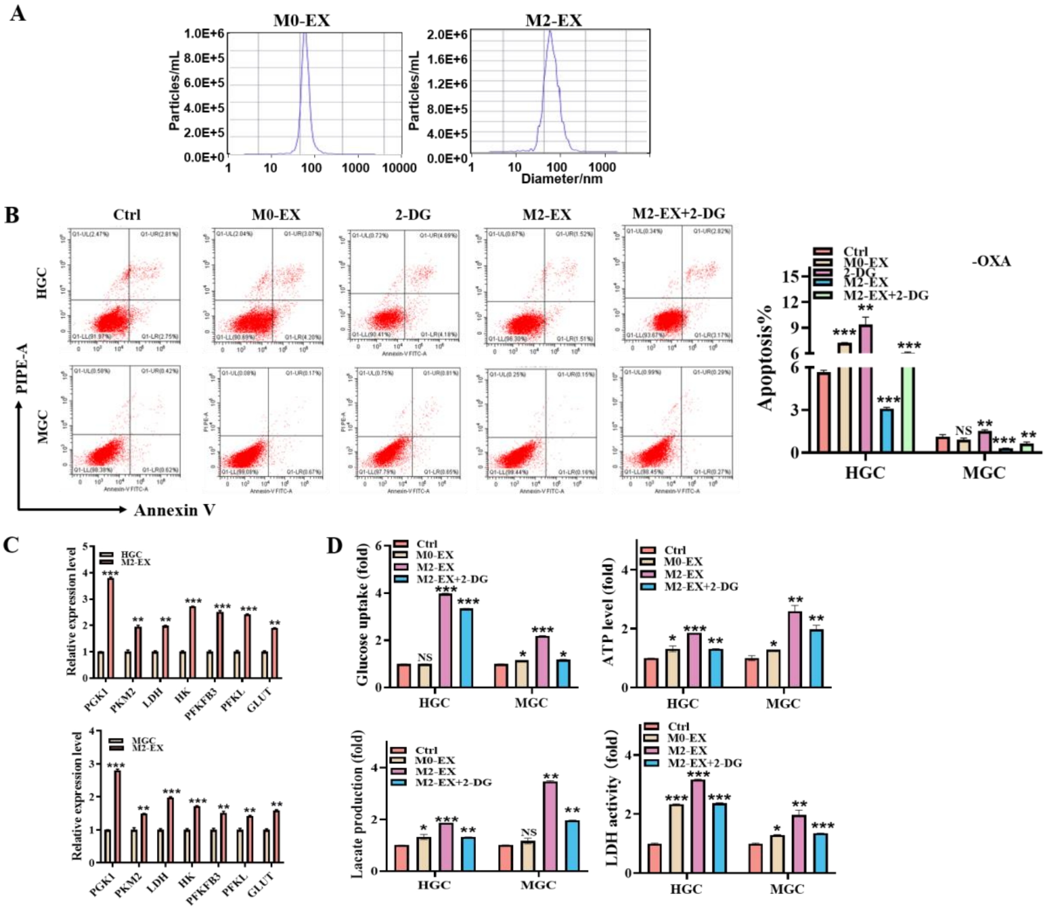


**Supplementary Figure 3. Characterization of M0-EX and M2-EX**

**A.** Nanoparticle tracking analyses of the size of M0-EX and M2-EX. **B.** Flow cytometric analyses of apoptosis in gastric cancer cells treated with M2-EX in the presence or absence of 2-DG. **C.** QRT-PCR analyses of glycolysis-related gene expression in gastric cancer cells treated with M2-EX. **D.** Glucose uptake, lactate production, ATP level, and LDH activity assays in gastric cancer cells treated with M2-EX. **$P< 0.01$, ***$P<0.001$.

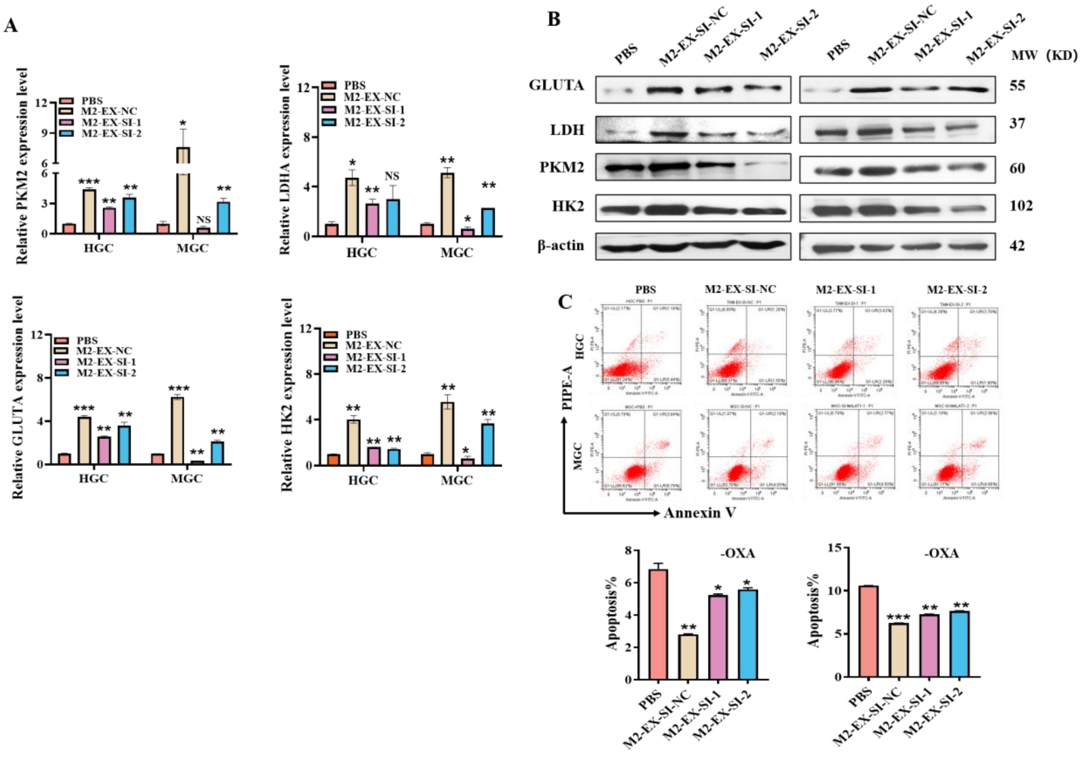


**Supplementary Figure 4. Identification and verification of M2-EX enriched lncRNAs**

**A-B.** QRT-PCR (A) and Western blot (B) analyses of glycolysis-related gene expression in gastric cancer cells treated with control or *MALAT1*-depleted M2-EX. **C.** Flow cytometric analyses of apoptosis in gastric cancer cells treated with control or *MALAT1*-depleted M2-EX. *$P$<0.05, **$P$< 0.01, ***$P$<0.001.

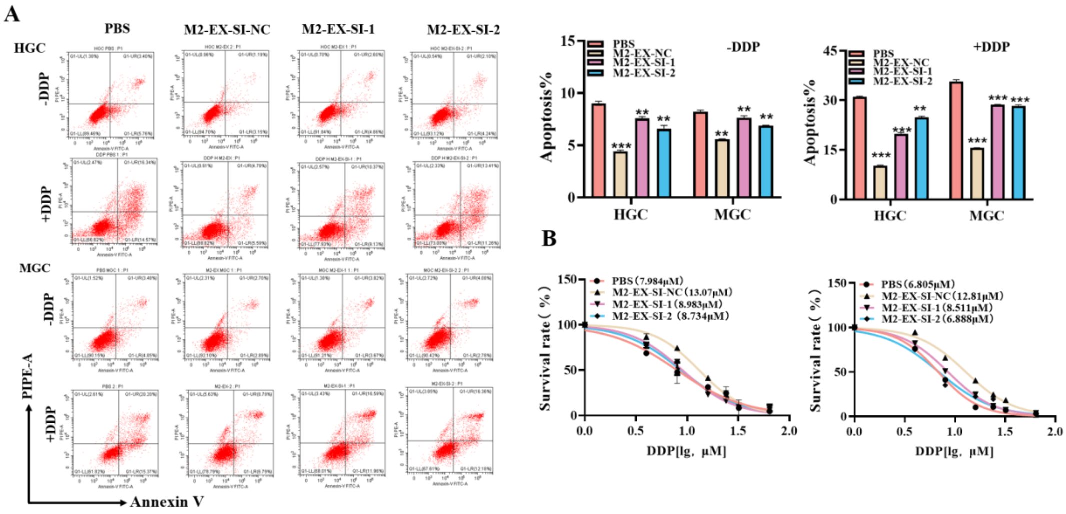


**Supplementary Figure 5. Exosomal *MALAT1* from M2-polarized macrophages induces resistance to cisplatin therapy in gastric cancer cells**

**A.** Flow cytometric analyses of cisplatin-induced apoptosis in gastric cancer cells treated with control or *MALAT1*-depleted M2-EX. **B.** CCK8 assay for IC50 of cisplatin in control or *MALAT1*-depleted M2-EX-treated gastric cancer cells. ***P*< 0.01, ****P*<0.001.

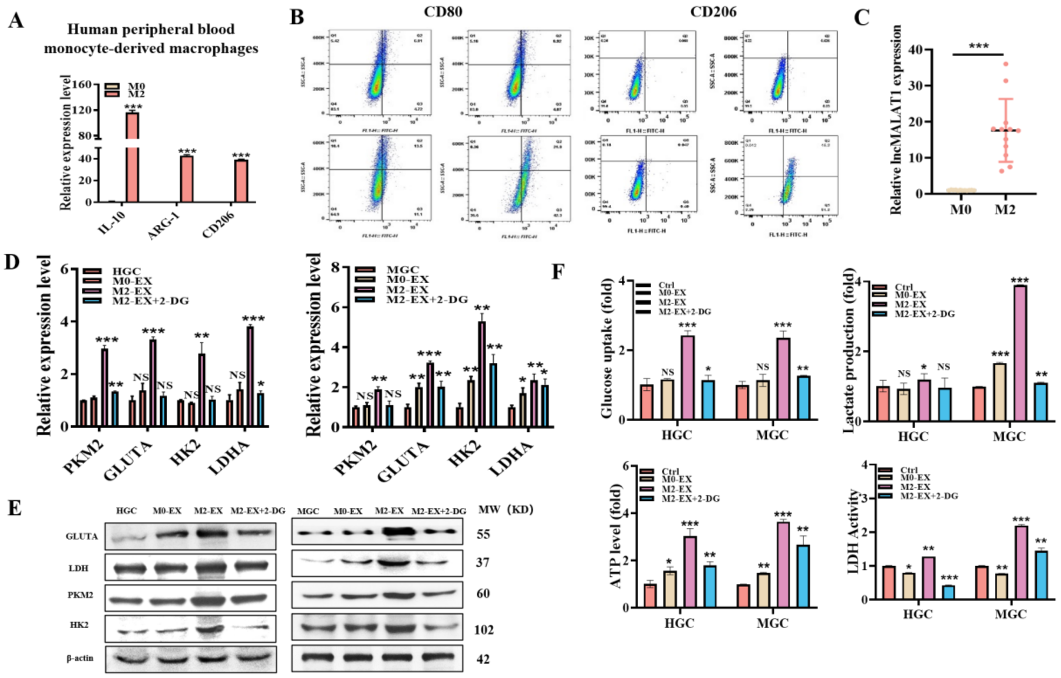


**Supplementary Figure 6. Exosomal *MALAT1* from M2-polarized macrophages induces gastric cancer cell glycolysis**

**A.** QRT-PCR analyses of arginase1, IL-10, and CD206 expression in M2-polarized macrophages from human peripheral blood monocytes. **B.** Flow cytometric analyses of CD206 expression in M2-polarized macrophages from human peripheral blood monocytes. **C.** QRT-PCR analyses of *MALAT1* expression in M2-polarized macrophages from human peripheral blood monocytes. **D-E.** QRT-PCR (D) and Western blot (E) analyses of glycolysis-related gene expression in gastric cancer cells treated with exosomes from M2-polarized macrophages from human peripheral blood monocytes. **F.** Glucose uptake, lactate production, ATP level, and LDH activity assays for gastric cancer cells treated with exosomes from M2-polarized macrophages from human peripheral blood monocytes. *$P$<0.05, **$P$< 0.01, ***$P$<0.001.

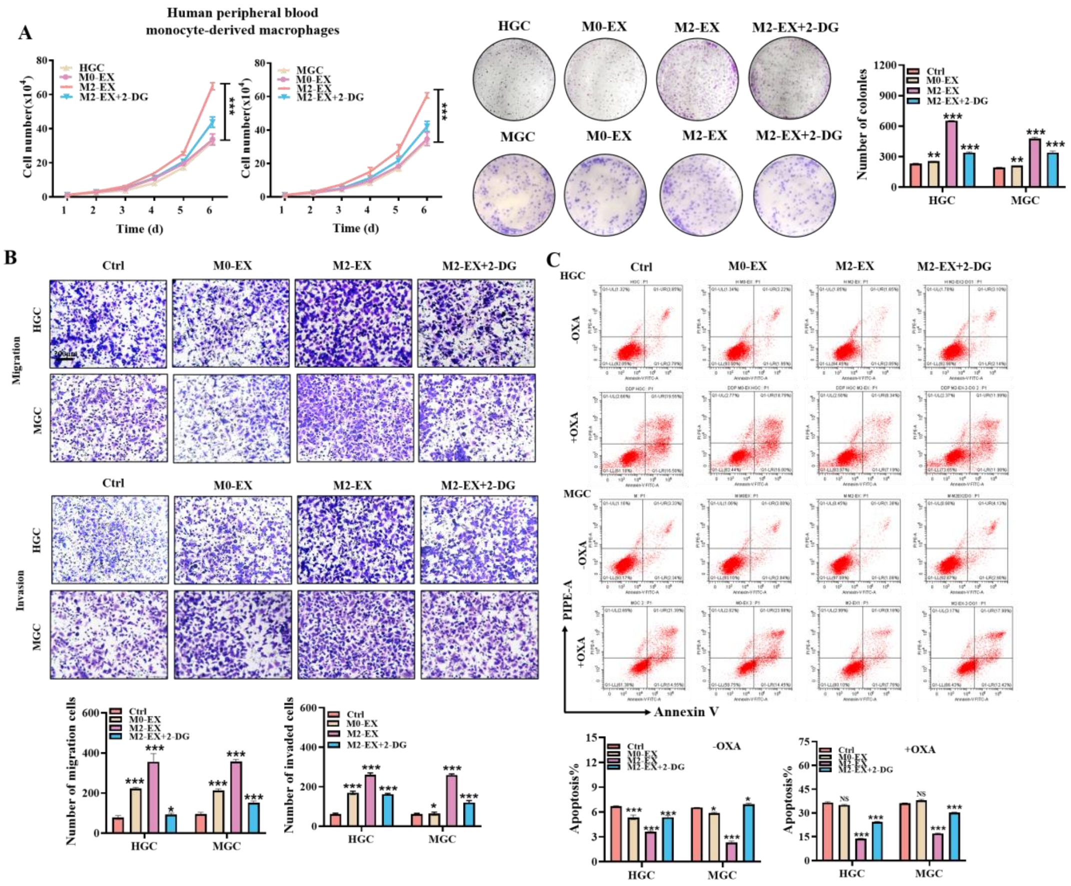

**Supplementary Figure 7. Exosomal *MALAT1* from M2-polarized macrophages from human peripheral blood monocytes promotes gastric cancer progression**

The roles of exosomes from M2-polarized macrophages from human peripheral blood monocytes in gastric cancer cell proliferation, migration, invasion, and chemoresistance were analyzed by cell counting and colony formation (A), transwell migration, and Matrigel invasion (B) assays and flow cytometric analyses (C).Scale bar: 200μm. **P*<0.05, ***P*< 0.01, ****P*<0.001.

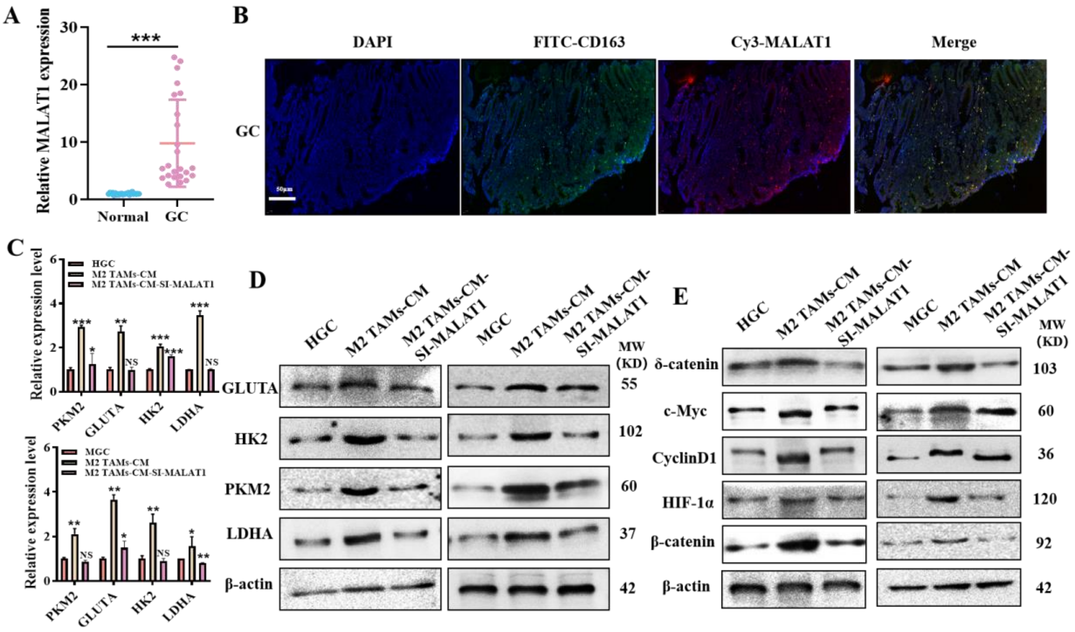


**Supplementary Figure 8. MALAT1 is upregulated in M2 TAMs from human gastric cancer tissue**

A. Normal and gastric cancer tissue derived macrophages were separated by CD14+ magnetic beads. The expression level of *MALAT1* was determined by qRT-PCR. B. Representative immunofluorescence staining for the co-localization of *MALAT1* and in gastric cancer tissues. Scale bar: 50μm. C-D. QRT-PCR (C) and western blot (D) analyses of glycolysis-related gene expression in gastric cancer cells treated with conditioned medium from human gastric cancer tissues derived M2 macrophages (M2-CM). *$P$<0.05, **$P$< 0.01, ***$P$<0.001.

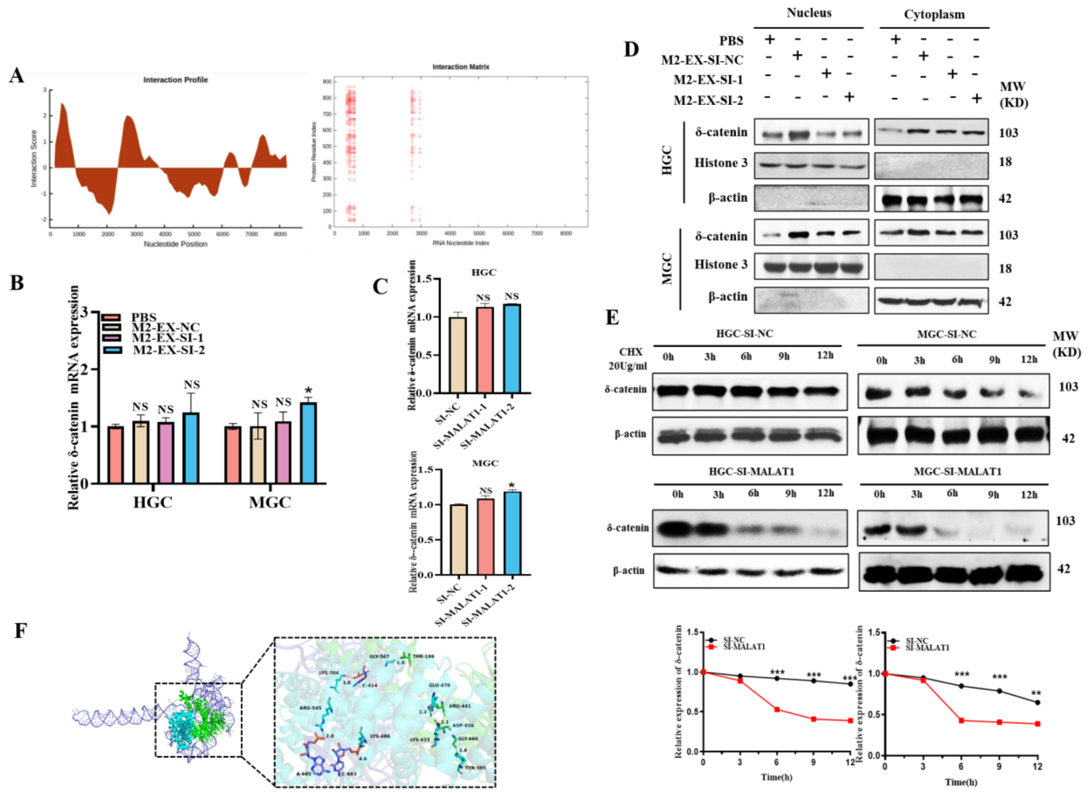


**Supplementary Figure 9. Exosomal *MALAT1* from M2-polarized macrophages stabilizes the δ-catenin protein and activates the β-catenin pathway**

**A.** Bioinformatic analyses of the potential binding sites for *MALAT1* and β-TRCP in the δ-catenin protein. **B.** QRT-PCR analyses of δ-catenin gene expression in the gastric cancer cells treated with M2-EX. **C.** QRT-PCR analyses of δ-catenin gene expression in the gastric cancer cells treated with *MALAT1* siRNA. **D.** CHX assay for the half-life of δ-catenin protein in gastric cancer cells treated with *MALAT1* siRNA. **E.** Nuclear and cytoplasmic localization of δ-catenin in the gastric cancer cells treated with control or *MALAT1*-depleted M2-EX. **F.** Molecular docking for *MALAT1* (purple), β-TRCP (green), and δ-catenin (blue) proteins by GRAMM and AutoDockTools. *$P$<0.05, **$P$<0.01, ***$P$<0.001.

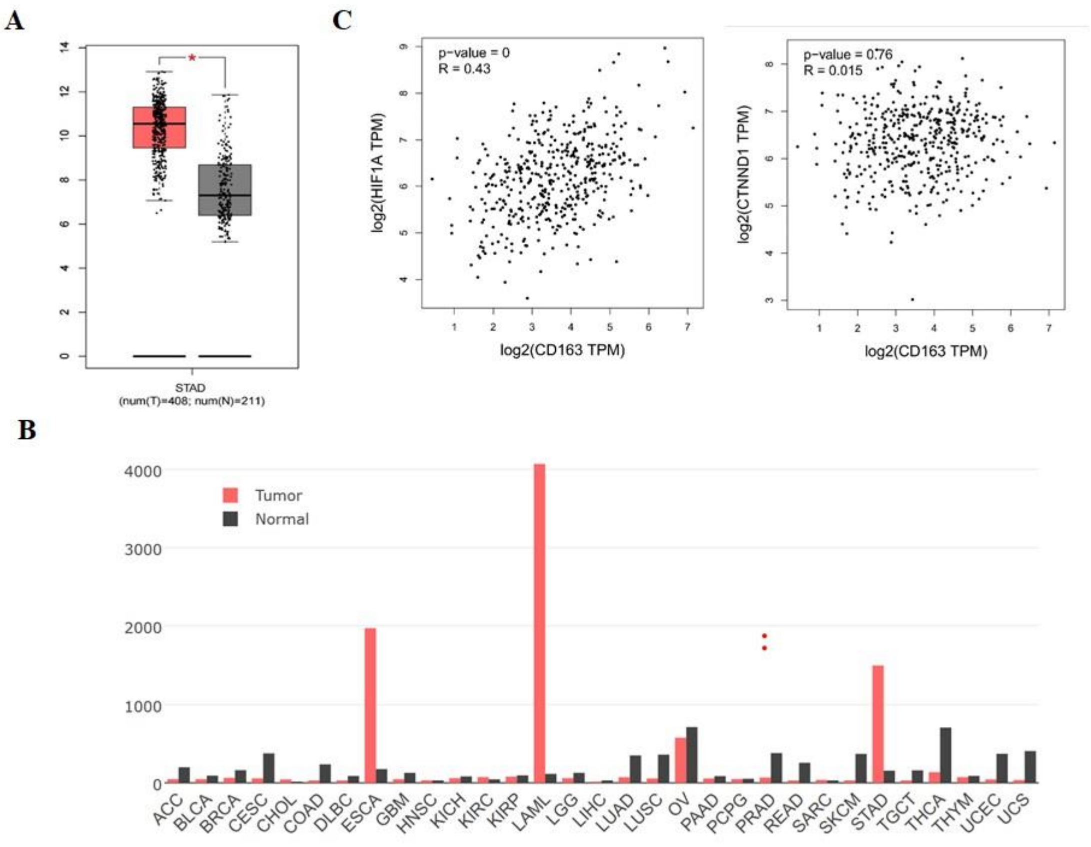


**Supplementary Figure 10. *MALAT1* is upregulated in gastric cancer and high infiltration of macrophages is correlated with upregulated HIF-1α expression.**

**A-B.** TCGA (A) and GEPIA (B) data analyses of *MALAT1* expression in gastric cancer tissues and normal tissues. **C.** TCGA data analyses of the correlation between CD163 and HIF-1α gene expressions in gastric cancer tissues. *$P$<0.05.

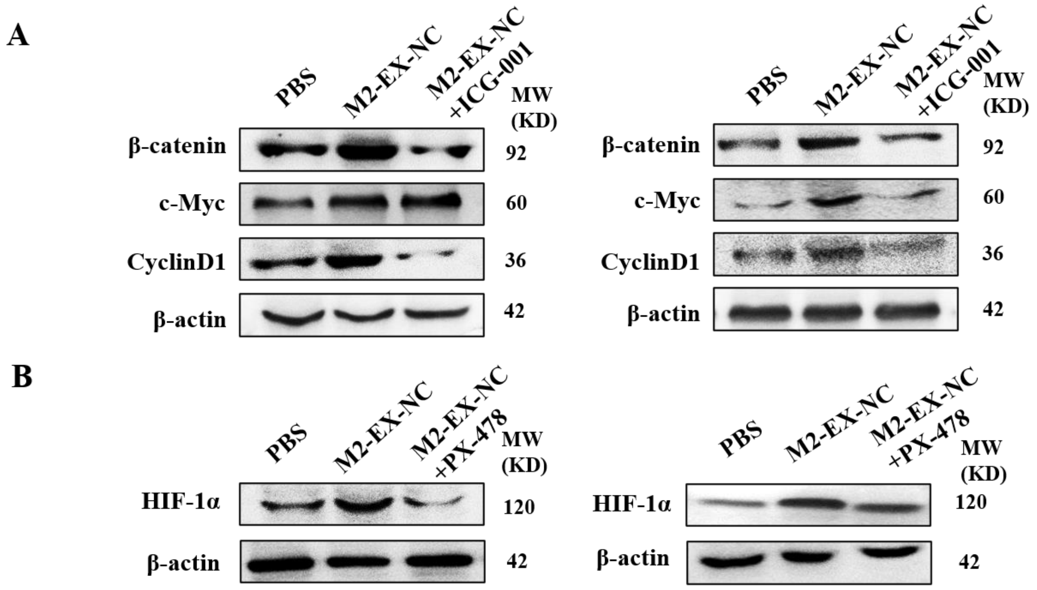


**Supplementary Figure 11. The effects of ICG001 and PX-478 on β-catenin and HIF-1α signaling pathways**

Western blot analyses of the expression of β-catenin (A) and HIF-1α (B) in gastric cancer cells treated with M2-EX in the presence or absence of ICG001 and PX-478.

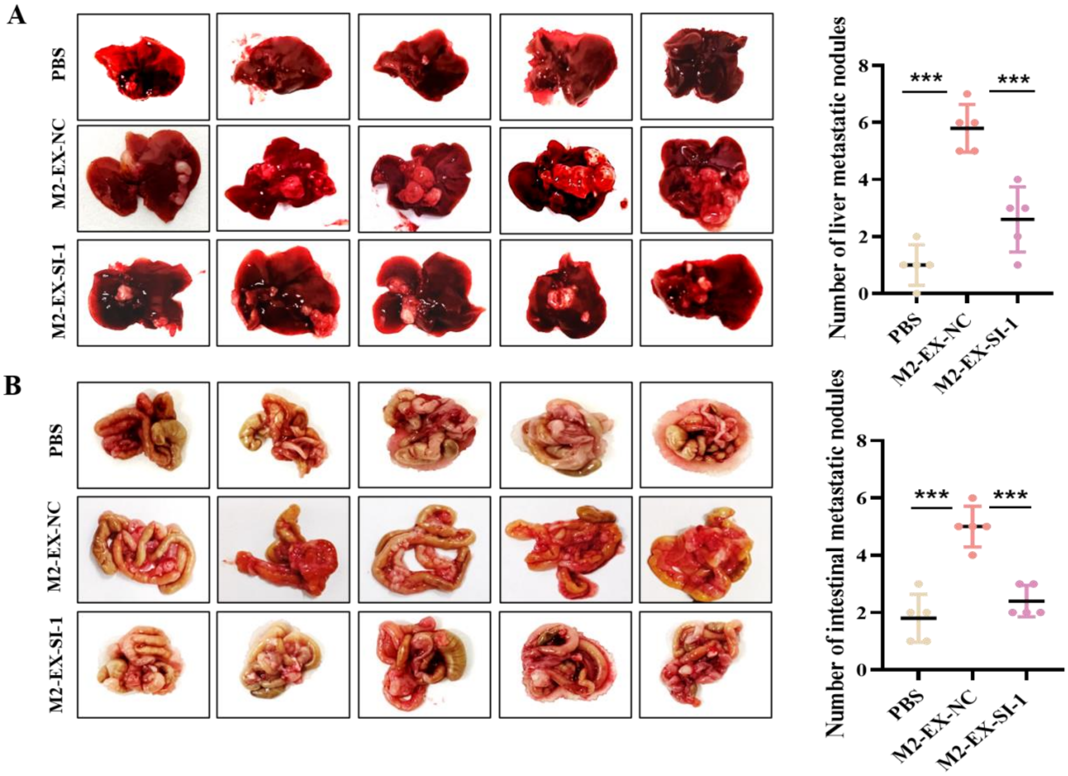


**Supplementary Figure 12. The effects of exosomal *MALAT1* from M2-polarized macrophages on gastric cancer metastasis**

The metastatic nodules in the livers (A) and colon tissues (B) in different groups of mouse tumor models as indicated. ****P*<0.001.

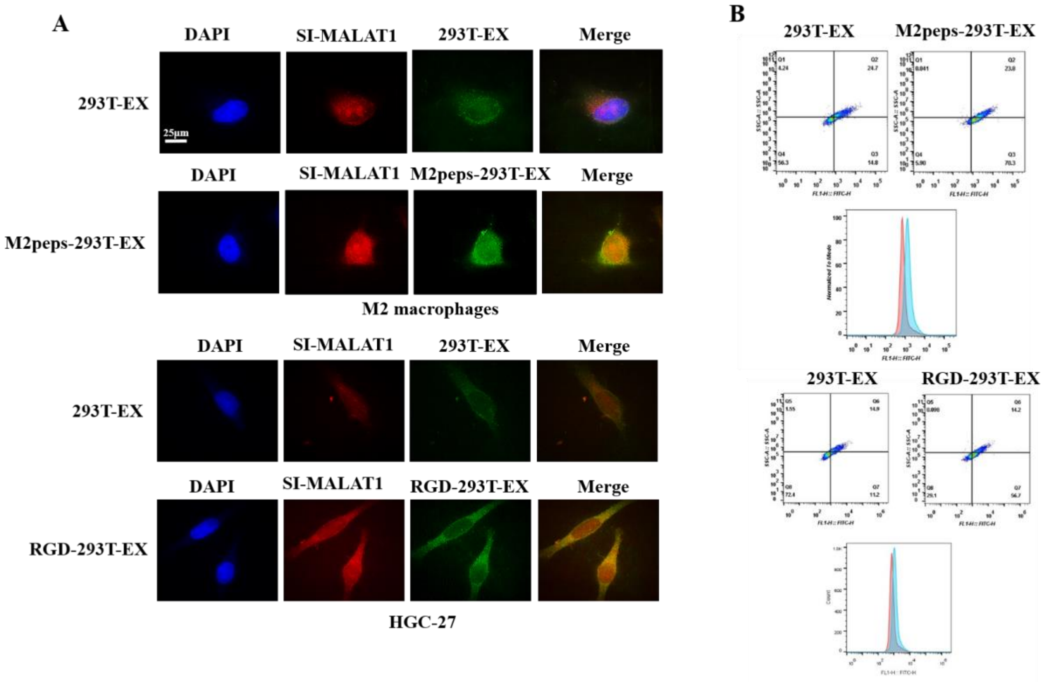


**Supplementary Figure 13. The internalization of RGD- or M2pep-decorated and si-*MALAT1*-encapsulated exosomes**

**A.** Cellular uptake assay for RGD- or M2pep-decorated and si-*MALAT1*-encapsulated exosomes by gastric cancer cells and M2 macrophages. Scale bar: 25μm. **B.** Flow cytometric analyses of the internalization efficiency.

**Table S1 The sequences of primers for qRT-PCR**

| Gene | Sequence | Temperature ($^oC$) |
|---|---|---|
| β-actin | F: 5’-CACGAAACTACCTTCAACTCC-3’<br>R: 5’-CATACTCCTGCTTGCTGATC-3’ | 56 |
| CD206 | F:5'-CCATCGAGGAAGAGGTTCGG-3'<br>R:5'-GGTGGGTTACTCCTTCTGCC-3' | 56 |
| ARG-1 | F:5'-ACCTGCCCTTTGCTGACATC-3'<br>R:5'-ACCAGGCTGATTCTTCCGTT-3' | 58 |
| IL-10 | F:5'-CCAAGACCCAGACATCAAGG-3' R:5'-<br>GCATTCTTCACCTGCTCCAC-3' | 56 |
| *MALAT1* | F:5'-GGGTGTTTACGTAGACCAGAACC-3' R:5'-<br>CTTCCAAAAGCCTTCTGCCTTAG-3' | 56 |
| HIF-1α | F:5'-TCCATTTTCAGCTCAGGACACT-3' R:5'-<br>GGTAGGTTTCTGTAACTGGGTCT-3' | 58 |
| GLUTA | F:5'-CTTTGTGGCCTTCTTTGAAGTG-3'<br>R:5'-GACCACACAGTTGCTCCACATAC-3' | 58 |
| HK-2 | F:5'-GCCATCCTGCAACACTTAGGGCTTGAG-3'<br>R:5'-GTGAGGATGTAGCTTGTAGAGGGTCCC-3' | 58 |
| LDHA | F:5'-ATGGCAACTCTAAAGGATCA-3'<br>R:5'-GCAACTTGCAGTTCGGGC-3' | 58 |
| PKM2 | F:5'-GCCCGTGAGGCAGAGGCTGC-3'<br>R:5'-TGGTGAGGACGATTATGGCCC-3' | 58 |
| CyclinD1 | F:5'-CCGAGAAGCTGTGCATCTAC-3'<br>R:5'-CTTCACATCTGTGGCACAGAG-3' | 60 |
| c-Myc | F:5'-GGCTCCTGGCAAAAGGTCA-3'<br>R:5'-CTGCGTAGTTGTGCTGATGT-3' | 58 |
| δ-catenin | F:5'-ATGTTTGCGAGGAAGCCGC-3'<br>R:5'-CGAGTGGTCCCATCATCTG-3' | 58 |
